# YdiV regulates *Escherichia coli* ferric uptake by manipulating the DNA-binding ability of Fur in a SlyD-dependent manner

**DOI:** 10.1093/nar/gkaa696

**Published:** 2020-08-19

**Authors:** Fengyu Zhang, Bingqing Li, Hongjie Dong, Min Chen, Shun Yao, Jingwen Li, Honghai Zhang, Xiangguo Liu, Hongwei Wang, Nannan Song, Kundi Zhang, Ning Du, Sujuan Xu, Lichuan Gu

**Affiliations:** State Key Laboratory of Microbial Technology, Shandong University, 72 Binhai Road, Qingdao 266237, P.R. China; Key Laboratory of Rare and Uncommon Diseases, Department of Microbiology, Institute of Basic Medicine, Shandong First Medical University & Shandong Academy of Medical Sciences, 18877 Jingshi Road, Jinan 250062, P.R. China; State Key Laboratory of Microbial Technology, Shandong University, 72 Binhai Road, Qingdao 266237, P.R. China; State Key Laboratory of Microbial Technology, Shandong University, 72 Binhai Road, Qingdao 266237, P.R. China; School of Life Sciences, Shandong University, 72 Binhai Road, Qingdao 266237, P.R. China; Qingdao Institute of Bioenergy and Bioprocess Technology, Chinese Academy of Sciences, 189 Songling Road, Qingdao 266237, P.R. China; Key Laboratory of Infection and Immunity of Shandong Province and Department of Immunology, School of Basic Medical Sciences, Shandong University, 44 Wenhuaxi Road, Jinan 250012, P. R. China; School of Life Sciences, Shandong University, 72 Binhai Road, Qingdao 266237, P.R. China; State Key Laboratory of Microbial Technology, Shandong University, 72 Binhai Road, Qingdao 266237, P.R. China; Key Laboratory of Rare and Uncommon Diseases, Department of Microbiology, Institute of Basic Medicine, Shandong First Medical University & Shandong Academy of Medical Sciences, 18877 Jingshi Road, Jinan 250062, P.R. China; State Key Laboratory of Microbial Technology, Shandong University, 72 Binhai Road, Qingdao 266237, P.R. China; School of Life Sciences, Shandong University, 72 Binhai Road, Qingdao 266237, P.R. China; State Key Laboratory of Microbial Technology, Shandong University, 72 Binhai Road, Qingdao 266237, P.R. China; State Key Laboratory of Microbial Technology, Shandong University, 72 Binhai Road, Qingdao 266237, P.R. China

## Abstract

Iron is essential for all bacteria. In most bacteria, intracellular iron homeostasis is tightly regulated by the ferric uptake regulator Fur. However, how Fur activates the iron-uptake system during iron deficiency is not fully elucidated. In this study, we found that YdiV, the flagella gene inhibitor, is involved in iron homeostasis in *Escherichia coli*. Iron deficiency triggers overexpression of YdiV. High levels of YdiV then transforms Fur into a novel form which does not bind DNA in a peptidyl-prolyl cis-trans isomerase SlyD dependent manner. Thus, the cooperation of YdiV, SlyD and Fur activates the gene expression of iron-uptake systems under conditions of iron deficiency. Bacterial invasion assays also demonstrated that both *ydiV* and *slyD* are necessary for the survival and growth of uropathogenic *E. coli* in bladder epithelial cells. This reveals a mechanism where YdiV not only represses flagella expression to make *E. coli* invisible to the host immune system, but it also promotes iron acquisition to help *E. coli* overcome host nutritional immunity.

## INTRODUCTION

The transition metal iron is essential for bacterial survival and growth. As an indispensable cofactor, iron is involved in many cellular processes, including N_2_ fixation, DNA synthesis, and respiration (1). However, although iron is the fourth most abundant element in the Earth's crust, iron limitation is a challenge for bacteria due to the extremely low solubility (10^−18^ M at pH 7.0) of the Fe^3+^ ion (2). For pathogenic bacteria, this challenge is even more severe since humans and other mammals have evolved a complicated mechanism of nutritional immunity to restrict iron availability (3–5). Mechanisms of nutritional immunity include, but are not limited to, heme-Fe sequestration, siderophore-Fe sequestration, ferritin-Fe storage, lactoferrin-Fe^3+^ combination, oxidation of Fe^2+^ to insoluble Fe^3+^, and macrophage protein 1 dependent Fe^2+^ export protein (6–9). As a result, the concentration of free iron in the plasma of humans (∼10^−24^ M) is much lower than that required for bacterial growth (∼10^−6^ M) (3,10). Consequently, pathogenic bacteria must overcome the nutritional immunity to successfully establish infection (11).

*Escherichia coli* (pathogenic or nonpathogenic strains) have evolved many strategies to acquire sufficient iron from their environment or host. Synthesis of enterobactin (Ent), a catecholate type of siderophore, is a dominant strategy used by *Escherichia coli* to salvage iron (12–14). Ferric-Ent is recognized by FepA, an outer membrane receptor, and transported into the periplasm in a TonB-dependent process. Once in the periplasm, ferric-Ent is immediately captured by FepB and then delivered by the ATP-binding cassette (ABC) transporter, FepDGC, into the cytoplasm where the Fe-Ent can be utilized (8,15,16). Intriguingly, iron is not only necessary, but it is also dangerous for bacteria. A high intracellular Fe (II) concentration can trigger a Fenton reaction, causing cell toxicity (17). Therefore, bacteria must keep intracellular iron levels at appropriate concentrations in order to satisfy their physiological needs while avoiding harm.

When intracellular Fe^2+^ is low, *E. coli* increases Ent production to sequester more iron (18,19). When intracellular iron is high, *E. coli* reduces Ent production and may also pump iron out of cell (20). Regulation of the intracellular iron homeostasis is dependent upon the ferric uptake regulator (Fur) protein (21). Fur is a dimeric protein that is highly conserved across many bacteria. The Fur monomer binds a structural Zn^2+^ and has a regulatory binding site for the Fe^2+^. The mechanism of Fur in ferric regulation has been well established. Under iron-sufficient conditions, Fe^2+^ binds to the regulatory site, and the Fur homodimer then combines with the operator site of a target promoter, blocking the binding of the RNA polymerase holoenzyme (RNAP) and inhibiting iron-uptake genes expression (22). While under iron-limited conditions, Fur releases the regulatory Fe^2+^ and dissociates from the target DNA sequence, thus relieving the iron-uptake gene repression.

Flagella also play important roles in the invasion of pathogenic bacteria, but it is also a strong signal of danger to the host immune system (23,24). In order to successfully invade and avoid recognition by the host's innate immune system, pathogenic bacteria must precisely control their flagella expression levels during adhesion, invasion, and colonization (25). As such, it no surprise that recent studies have found that the regulation of iron homeostasis and flagellar biogenesis are coordinated in some bacteria. Transcriptomic analyses and ChIP-chip assays have revealed that *Campylobacter jejuni* Fur protein is associated with iron acquisition, oxidative stress defense, flagellar biogenesis, and energy metabolism (26–28). Similarly, the *Helicobacter pylori* Fur regulon also includes several genes involved in flagella biogenesis (29). In *H. pylori* J99, Fur has been shown to positively modulate motility through interfering with bacterial flagellar switching (30). Moreover, flagellar gene transcription is inhibited immediately upon nutrient starvation (31). Recently, it was also reported that in uropathogenic *E. coli* (UPEC) Fur suppresses the expression of type 1 fimbriae and flagella genes when grown under iron-rich conditions, but disinhibit these genes under iron-restricted conditions, such as in patients with urinary tract infections (32).

All of these studies suggest that regulation of iron homeostasis and flagellar biogenesis are highly coordinated. However, the underlying mechanism is unknown. This is especially true for intracellular pathogens, such as UPEC. Infection of UPEC generally contains four stages: adhesion, invasion, intracellular bacterial community (IBC) formation, and dispersal (fluxing) from the intracellular environment (33). During this process, bacteria move from an environment with a relatively high-iron concentration (about 10^−6^ M in the urine) to an intracellular, iron-starvation environment (12,34–36). Additionally, the bacteria transform from a motile state into a sessile state, since flagellum-mediated motility is needed for adhesion and invasion, but flagella need to be shut down throughout early stages of IBC development as a way to evade the innate immune system (37). This process requires the regulation of iron acquisition genes and flagella genes, which have opposite phases, i.e., the former has an increase while the later has a decrease. In this regard, Fur cannot accomplish this task since it synchronously regulates iron acquisition and flagella biogenesis. Thus, there must be some crosstalk between Fur and classic flagella regulatory mechanisms.

In *E. coli*, the expression of flagella is tightly controlled by the master regulator FlhD_4_C_2_ complex. The transcriptional function of FlhD_4_C_2_ is inhibited by YdiV, a degenerate EAL domain protein, in a concentration-dependent manner. This negatively regulates flagella biogenesis and, thus, bacterial motility (38–40). Interestingly, YdiV is induced under nutrient-starvation conditions (41), and its homologue is highly upregulated during invasion of *Salmonella* (42), which suggests its important role in bacterial infection.

Here, we report that YdiV is upregulated during iron starvation in *E. coli*. High levels of YdiV transform Fur into a novel form that does not have DNA-binding activity, and as a result, it activates the gene expression of iron-uptake systems during iron deficiency. This interaction is acted in a peptidyl-prolyl cis-trans isomerase SlyD-dependent manner. YdiV and SlyD play their role cooperatively by switching the folding path of Fur and are necessary for survival and growth of UPEC in bladder epithelial cells (BECs). This suggests that YdiV is a dominant regulator over Fur and reveals a novel mechanism where YdiV not only represses flagella expression in order to make *E. coli* invisible to the host immune system, but it also promotes iron acquisition to help *E. coli* overcome host nutritional immunity.

## MATERIALS AND METHODS

### Bacterial strains, plasmids and culture conditions

Bacterial strains used in this study are listed in Supplementary Table S1. The gene-deficient mutants were obtained through gene knockout by using the λ-Red mediated recombinase system described previously (43). Primers used for gene knockout are listed in Supplementary Table S2. For the construction of MG1655 Δ*ydiV*, MG1655 Δ*slyD* and MG1655 Δ*fur* strains, the corresponding primers in addition to template plasmid pKD4 (43) were used to obtain linear DNA fragments with kanamycin gene cassettes flanked by FRT (FLP recognition target) sites and homologous arms. Polymerase chain reaction (PCR) products were transformed into *E. coli* MG1655 cells harboring the helper plasmid pTKRED (44) by electroporation (Eppendorf Electroporator). Positive clones were confirmed by PCR test (Supplementary Table S2), and the resistance gene was removed (45). BL21 Δ*ydiV*, BL21 Δ*slyD*, BL21 Δ*fur*; UPEC Δ*ydiV* and UPEC Δ*slyD* strains were also constructed using the same method, except *E. coli* BL21(DE3) or UPEC CFT073 were used as the parent strain.

Plasmids used in this study were constructed using the Gibson assembly method (46) and are listed in Supplementary Table S3. Linearized plasmid vectors and the desired genomic DNA fragments to be overexpressed were amplified by PCR using Phusion High Fidelity DNA polymerase (New England Biolabs) and their corresponding primers (Supplementary Table S4). The reaction mixture containing linearized plasmid, genomic DNA fragments, and Gibson Assembly Master Mix (New England Biolabs) was then incubated at 50°C for 1 hour, and the ligated plasmid was then transformed into *E. coli* DH5α. Positive clones were further verified by DNA sequencing. Finally, the plasmid DNA was extracted using Plasmid Mini Kit (Omega) and saved at -20°C for future use. The time course induction of *ydiV* from pTrac*ydiV* (induced by IPTG) and pBAD*ydiV* (induced by arabinose) plasmids was measured in the Δ*ydiV* strain by using real-time quantitative PCR (qRT-PCR) (Supplementary Figure S1).

Fur mutant plasmids were constructed using the improved QuikChange method (47). Partially overlapping primers (Supplementary Table S4), in addition to template plasmid pGL01*fur*, were used to obtain Fur P18A and Fur P29A mutated linear DNA fragments through PCR. After transformation, *E. coli* DH5α repaired the breaks to yield the plasmid with the desired mutation.

*Escherichia coli* MG1655 and UPEC CFT073 were propagated in LB medium (1 l: 10 g tryptone, 5 g yeast extract, and 10 g NaCl) or M9 medium (1 l: 15 g Na_2_HPO_4_▪12H_2_O, 3 g KH_2_PO_4_, 0.5 g NaCl, 1 g NH_4_Cl, 30 ml 20% (v/v) glucose, 1 ml 0.1 M CaCl_2_, 1 ml 1 M MgSO_4_ and 1 ml vitamin mixture) at 37°C with shaking at 200 rpm. When necessary, antibiotics were added at the following concentrations: ampicillin 100 μg/ml, kanamycin 50 μg/ml, chloramphenicol 17 μg/ml, and spectinomycin 50 μg/ml. We added 0.3 mM IPTG or 1 mg/ml l-arabinose as inducers.

For iron deficiency-induced cultures, the strains were activated in LB medium containing the appropriate antibiotics over 10 h. Then the above cells were diluted to an OD_600_ of 0.05 in fresh medium for growth. When the strains entered mid-log phase, 200 μM of 2,2′-dipyridyl (Sigma-Aldrich) and IPTG or l-arabinose (as needed) were added to achieve an iron deficient condition and induce *ydiV* expression. The cultures were sampled at different times after the addition of the iron chelator and stored at −80°C for subsequent RNA isolation. For the corresponding iron-sufficient group, the culture conditions were consistent with the iron-deficient group, except 2,2′-dipyridyl was not added.

### RNA isolation and real-time quantitative PCR (qRT-PCR)

Total RNA was extracted using the MiniBEST Universal RNA Extraction Kit (TaKaRa) according to manufacturer's instruction. The PrimeScript RT reagent Kit (TaKaRa) was used for cDNA synthesis. Primers for qRT-PCR are listed in Supplementary Table S5. The qRT-PCR reactions were performed on a QuantStudio^TM^ Design & Analysis Software 1.3.1 (Thermo Fisher Scientific) using SYBR Premix Ex Taq™ II Kit (TaKaRa). The expression of *gapA* mRNA was used to normalize the target gene expression (42). The relative transcript abundance was calculated using the 2^−ΔΔCt^ method (48).

### β-Galactosidase assay

For β-galactosidase assays, the *ydiV* gene from *E. coli* MG1655 was subcloned into an arabinose-induced pBAD24 vector. The reporting plasmids pCL *fepAp-lacZ* and pCL *fhuFp-lacZ* were also constructed using the Gibson assembly method (46) from three fragments: *fepA* promoter or *fhuF* promoter, *E. coli lacZ* DNA fragment and the promoter-removed linear pCL1920 vector (primers used for amplification of the fragments are listed in Supplementary Table S4). After construction, plasmids pCL *fepAp-lacZ* or pCL *fhuFp-lacZ*, pBAD24*ydiV* or empty vector pBAD24 were transformed into MG1655, Δ*ydiV*, or Δ*fur* strains for β-galactosidase activity assays.

The target strain was inoculated into 50 ml LB medium containing ampicillin and spectinomycin at 30°C 200rpm, and cultured to OD_600_ = 0.2. Then 0.8 mg/ml of arabinose and 200 μM 2,2′-dipyridyl was added into the culture induce *ydiV* gene expression. Then 1 ml of the culture medium was collected at different induction times to measure both the OD_600_ and β-galactosidase activity. For the iron-sufficient group, all conditions were the same, except 2,2′-dipyridyl was not added. The reagent preparation, sample processing and β-galactosidase activity detection were finished using the Miller method (49,50) and measured on a Synergy 4 Microplate Reader (BioTek Synergy HT).

### Protein expression and purification

The native Fur protein was purified from *E. coli* BL21 harboring the pGL01*fur* plasmid. Fur^YdiV^ protein was purified from *E. coli* BL21 harboring the pGL01*fur* and pACYC*ydiV* plasmids. Fur^SlyD^ protein was purified from *E. coli* BL21 harboring the pGL01*fur* and pET29b*slyD* plasmids. Fur^YdiV, SlyD^ protein was purified from *E. coli* BL21 harboring the pGL01*fur*, pACYC*ydiV*, and pET29b*slyD* plasmids. *E. coli* BL21 Δ*ydiV* or BL21 Δ*slyD* strain was used instead of *E. coli* BL21 as needed. Fur mutants were expressed in the *E. coli* BL21 Δ*fur* strain. YdiV protein was purified from *E. coli* BL21 harboring the pGL01*ydiV* plasmid.

The strains were incubated in two liters of LB medium supplemented with the appropriate antibiotic at 37°C at 200 rpm and allowed to grow until an OD_600_ of 0.6. Then 0.2 mM IPTG was added to induce protein expression at 16°C. After 16 h of induction, the cells were harvested in lysis buffer (25 mM Tris–HCl, pH 8.0, 200 mM NaCl, 1 mM PMSF, and 20 μg/ml DNase 1) and lysed by sonication. The lysate was centrifuged at 28 500 × g for 50 min, and then the supernatant was loaded onto a Ni-NTA column (GE Healthcare) for affinity chromatography. After elution from the Ni-NTA column by elution buffer (25 mM Tris–HCl, pH 8.0, 100 mM NaCl and 250 mM imidazole), the sample was purified by size-exclusion chromatography by using Superdex 200 (GE Healthcare) in 10 mM Tris–HCl and 100 mM NaCl at pH 8.0. If necessary, the PreScission protease was used to remove the His-tag. The entire isolation process was performed at 4°C. Finally, SDS-PAGE was used to assess protein purity.

### Electrophoretic mobility shift assays (EMSAs)

Fluorophore 6-carboxy-fluorescein (FAM)-labeled double-stranded Fur box DNA (GATAATGATAATGATAATGATAATGATAATGA) (51) and random scrambled DNA (ScDNA) (ATGAACAAGAAGATTCATTCCCTGG) was obtained by mixing two reverse complementary single-stranded FAM DNA to 10 mM in annealing buffer (10 mM Tris–HCl, pH 7.5, 150 mM NaCl). The mixture was heated at 95°C for 10 min and slowly cooled to room temperature. For the EMSA, 25 nM of DNA was incubated with different protein samples in reaction buffer (10 mM Tris–HCl, pH 7.5, 1 mM MgCl_2_, 40 mM KCl, 0.1 mg/ml BSA, 5% (w/v) glycerol) at 37°C for 15 min (52). We added 100 μM MnCl_2_ to the reaction buffer when necessary. The samples were then analyzed using a native 5% polyacrylamide gel at 80 V for 80 minutes in 0.5× TBE buffer (46 mM Tris base, 46 mM boric acid, 1 mM EDTA pH 8.0) at 4°C. Light was avoided in this experiment. Imaging and data analysis were performed using a Typhoon Scanner (GE Healthcare) and Imagequant software (GE Healthcare).

### Fluorescence polarization (FP) measurements

The fluorophore FAM-labeled Fur box DNA was obtained in the same way as the DNA used in the EMSA. To measure the binding of FAM-Fur box DNA to Fur variants, 1 nM of DNA was incubated with increasing amounts of protein (gradient diluted 15 times from 100 μM protein) in reaction buffer (10 mM Tris–HCl and 75 mM NaCl, pH 7.5) at 37°C and protected from light for 15 min. We added 150 μM of MnCl_2_ in the reaction buffer when necessary. FP measurements were conducted on a Synergy 4 Microplate Reader (BioTek Synergy HT). All of the experiments were performed in triplicate. The curves were fitted to deduce binding affinities by GraphPad Prism 5 software (GraphPad).

### Determination of iron and zinc concentration via inductively coupled plasma mass spectrometry (ICP-MS)

Native Fur and Fur^YdiV^ protein were concentrated to 5 mg/ml in a buffer containing 10 mM Tris–HCl and 100 mM NaCl. The UPEC mutant strains were inoculated into 50 ml of LB medium or LB medium containing 200 μM 2,2′-dipyridyl and cultured to an OD_600_ of 1.0. The cells were then collected and washed twice using a PBS solution containing 5 mM EDTA to remove the ions from the medium. The cells were then washed twice using PBS to remove the EDTA. Finally, the cells were dried at 60°C overnight.

After weighing, sample was added to a pre-cleaned digestion flask. A solution of HNO_3_, H_2_SO_4_ and HClO_4_ was poured into the samples at a volume ratio of 5:3:2, respectively, and placed in a fume hood for 24 h. After this, a hot plate heated to approximately 200–250°C was used to heat the samples until digested to near desiccation. After cooling, the samples were diluted with 5 ml of pure deionized water and placed on a hot plate at 100°C until evaporated to near desiccation and then cooled. The sample was diluted with 20 ml of deionized water and filtered for final analysis using ICP-MS (Thermo XSERIES2). The ICP-MS experiment was based on the standard of the ‘National Food Safety Standard, Determination of Multi-Elements in Food (GB 5009.268-2016)’ regulated by China.

### Isothermal titration calorimetry (ITC)

ITC was performed with an isothermal titration calorimeter (Microcal ITC200). The native Zn_1_Fur and Zn_1_Fur^YdiV^ were prepared by dialyzing as previously mentioned (52). Proteins were diluted to 100 μM with reaction buffer (25 mM Tris–HCl, pH 6.8 and 100 mM NaCl) and titrated against the ion buffer (25 mM Tris–HCl, pH 6.8, 100 mM NaCl, and 1200 μM ZnCl_2_ or 500 μM FeSO_4_). The titration process and parameter settings were designed according to the previous study (53).

### Size-exclusion chromatography

Proteins were prepared at concentrations varying between 100 and 200 μM at 4°C and then subjected to size-exclusion chromatography using a Superdex 200 column (GE Healthcare) equilibrated in buffer (10 mM Tris–HCl, pH 8.0 and 100 mM NaCl). The 62 and 27 kDa proteins were used as markers to determine the oligomerization state of Fur. All images were processed using Origin 8.0 software (Originlab).

### Differential scanning calorimetry

The calorimetry scanning of proteins was performed with a VP-DSC MicroCalorimeter (Microcal) at a scan range of 20–110°C. Buffer (25 mM Tris–HCl, pH 8.0 and 100 mM NaCl) was used in the reference cell of the calorimeter. The native Fur and Fur^YdiV^ proteins were diluted with the buffer and used at a concentration of 100 μM. The *T*_m_ values for the samples were analyzed by using the standard MicroCal VP-DSC analysis software (Microcal).

### Circular dichroism (CD)

The protein samples were desalted and diluted to 50 μg/l with 10 mM Tris–HCl pH 8.0. The CD spectra were recorded using a JASCO (J-810) spectropolarimeter (Jasco) in a 1.0 cm quartz cell with the wavelength ranging from 190 nm to 250 nm at 25°C.

### Pull-down assay for SlyD identification

The pull-down assay was performed according to a previously published method (54). *Escherichia coli* BL21 harboring the pGL01*fur* (N-terminal His-tag) plasmid was cultured and loaded onto a Ni-NTA column as mentioned in the protein expression and purification section of the Materials and Methods. Then 10 ml of reaction buffer (25 mM Tris–HCl, pH 8.0 and 200 mM NaCl) was flowed through the Ni-NTA column to remove proteins that were bound nonspecifically. The outlet of the Ni-NTA column was closed, and then 3 ml of reaction buffer and 0.4 mg of PreScission proteases were added at 4°C for over 5 h to release the Fur protein from the Ni-NTA column. Then the outlet of the Ni-NTA column was then opened and the flowing samples were collected. After that, the His-tag and remaining nonspecifically bound proteins were trapped by Ni-NTA column, and Fur and the proteins Fur specifically interacted with were collected. After size-exclusion chromatography purification (Superdex 200), the sample was tested by SDS PAGE and prepared for high-resolution HPLC–MS/MS assays.

### Sample preparation and high-resolution HPLC–MS/MS

Before the assay, protein samples were purified though size-exclusion chromatography and evaluated by SDS-PAGE. Then in-gel digestions were performed to prepare the mass spectrum samples (55). For molecular weight determination, Fur and Fur^YdiV^ were desalted and diluted to 1 mg/ml in 10 mM Tris–HCl pH 8.0 buffer without trypsin digestion.

HPLC–MS/MS was performed on the Dionex UltiMate 3000 Rapid Separation (RSLC) system (Thermo Scientific) coupled with an ESI-Q-TOF mass spectrometer (Bruker Daltonics). Proteins were separated on an XBridge Protein BEH C4 Column (2.1 mm × 50 mm I.D., particle size 3.5 μm) at 40°C with a mobile phase system of 0.1% formic acid (Sigma) in Milli-Q filtered water (A) and 0.1% formic acid (Sigma) in acetonitrile (Fisher Scientific) (B). The following gradient program was applied at a flow rate of 0.3 ml/min: 0–5 min, 95% A + 5% B; 5–30 min, 95–5% A + 5–95% B; 30–35 min, 5% A + 95% B; 35–50 min, 5–95% A + 95–5% B; and 50–55 min, 95% A + 5% B. The HPLC–MS/MS analysis was performed by using OTOF control software (Bruker Daltonics), and the protein molecules were calculated by charge deconvolution via Data Analysis software (Bruker Daltonics).

### Measurement of protein sulfhydryls

A quick measurement of protein sulfhydryls was performed by using Ellman's reagent (5,5’-dithiobis(2-nitrobenzoic acid), DTNB) (Sigma-Aldrich) as mentioned in the literature (56). Proteins were diluted to 10 μM in 1 ml PBS buffer, and then 200 μl of buffer 8.2 (100 mM boric acid, 0.2 mM EDTA, pH 8.2), 20 μl of 10 mM cystamine dihydrochloride (Sigma-Aldrich) and 20μl of 10 mM DTNB were added to start the reaction. We also added 2% SDS to the reaction to accelerate the reaction. The OD_412_ of the samples was measured using the multifunctional microplate detection system (BioTek Synergy HT). The parameters were calculated according to the formula in the precious study (56).

### Bacterial two-hybrid assay

The bacterial two-hybrid experiment was based on the reconstitution of adenylate cyclase in *E. coli* (57). Through the analysis of the crystal structures of Fur homologues, a number of plasmids were rationally constructed for protein-protein interaction characterization: pKNT25-*slyD*, pUT18C-*fur*, pKNT25-*ydiV*, and pCH363-*ydiV*. Pairs of plasmids were then co-transformed into *E. coli* BTH101, and recombinant strains were selected on LB–X-Gal plates (LB agar supplemented with 100 μg/ml ampicillin, 50 μg/ml kanamycin, 40 μg/ml X-Gal and 0.5 mM IPTG). A β-galactosidase assay was carried out using the Miller method (49,50) on a Synergy 4 Microplate Reader (BioTek Synergy HT).

### NMR spectroscopy

The ^15^N-labeled Fur and Fur^YdiV,SlyD^ proteins were expressed in *E. coli* cultured in ^15^N M9 medium (1 liter: 15 g Na_2_HPO_4_▪12H_2_O, 3 g KH_2_PO_4_, 0.5 g NaCl, 1 g ^15^NH_4_Cl, 30 ml 20% (v/v) glucose, 1 ml 0.1 M CaCl_2_, 1 ml 1 M MgSO_4_, and 1 ml vitamin mixture) containing the corresponding antibiotic and purified following the previous procedure mentioned in the protein expression and purification section of the Methods. Two-dimensional ^1^H–^15^N heteronuclear single quantum coherence (HSQC) experiments were performed at 298 K on a Bruker Avance 600-MHz spectrometer (Bruker), equipped with a *z*-axis gradient, and a triple resonance, cryogenic probe. Samples were prepared in buffer containing 10 mM Tris–HCl, pH 6.8, 100 mM NaCl in a 5%/95% (v/v) D_2_O/H_2_O mixture. The concentration was ∼70 μM. The data were processed and analyzed using the NMRPipe software (Bruker) (58).

### *In vitro* transcription assay

*The in vitro* transcription assays were performed as reported in the literature (59). The *E. coli fepA* promoters were prepared by PCR using primers (forward primer: 5′-CACCATAACCCCATGTTTAC-3′; reverse primer: 5′-ATGTCCGCGCTTCCCACGGC-3′) and cloned into T vector. Then the universal primer M13F and M13R were used to amplify a 230-bp DNA product as an experimental template. The reactions were performed in transcription buffer (40 mM Tris–HCl, pH 8.0, 75 mM NaCl, 5 mM MgCl_2_, 12.5% glycerol, 2.5 mM DTT and 50 μg/ml BSA). We added 2 μM MnCl_2_ in reaction buffer of groups Zn_1_Fe_1_Fur and Zn_1_Fe_1_Fur^YdiV, SlyD^. Reaction mixture (20 μl) containing 50 nM RNAP holoenzyme, 250 nM σ70, 10 nM DNA and 500 nM of different Fur proteins were incubated for 10 min at 37°C for open complex formation. RNA synthesis was started by addition of 1.2 μl of NTP mixture (2 mM ATP, 2 mM CTP, 2 mM GTP and 2 mM [α-^32^P]UTP (0.036 Bq/fmol)) for 15 min at 37°C. The RNA transcripts were separated on 15% urea–polyacrylamide slab gels (19:1 acrylamide/bisacrylamide) in 90 mM Tris-borate (pH 8.0) and 0.2 mM EDTA and then analyzed by storage-phosphor Typhoon Scanner (GE Healthcare).

### Cell culture and bacteria invasion assay

The human bladder carcinoma cell line 5637 (ATCC, HTB-9; referred to hereafter as bladder epithelial cells (BECs)) was maintained at 37°C with 5% CO_2_ in RPMI 1640 medium (Gibco) containing 10% fetal bovine serum (GE Healthcare Life Sciences). UPEC CFT073 and the mutant strains were grown for 10 h in LB medium at 37°C prior to infection of BECs. Bacteria were diluted to the same concentration using RPMI 1640 medium and then seeded on 5637 cells grown in 96-well plates at a multiplicity of infection of 200. After 1 h of infection, the bacteria were removed from plates. Then cells were washed twice with PBS, and then RPMI 1640 medium containing 200 μg/ml gentamicin was added for 2 h to kill the remaining extracellular bacteria. Cells were incubated in gentamicin-containing medium (10 μg/ml) for an additional 10 h (referred to as 12 h post-infection (hpi)) or 22 h (24 hpi). In order to count the invading bacteria, cells were washed gently with PBS and then disrupted with 1% Triton X-100 (Sigma-Aldrich) to release bacteria. Finally, serial dilutions of bacteria were plated on LB agar and colony forming units (CFUs) were counted.

## RESULTS

### YdiV regulates iron homeostasis in *E. coli*

**Figure 1.**
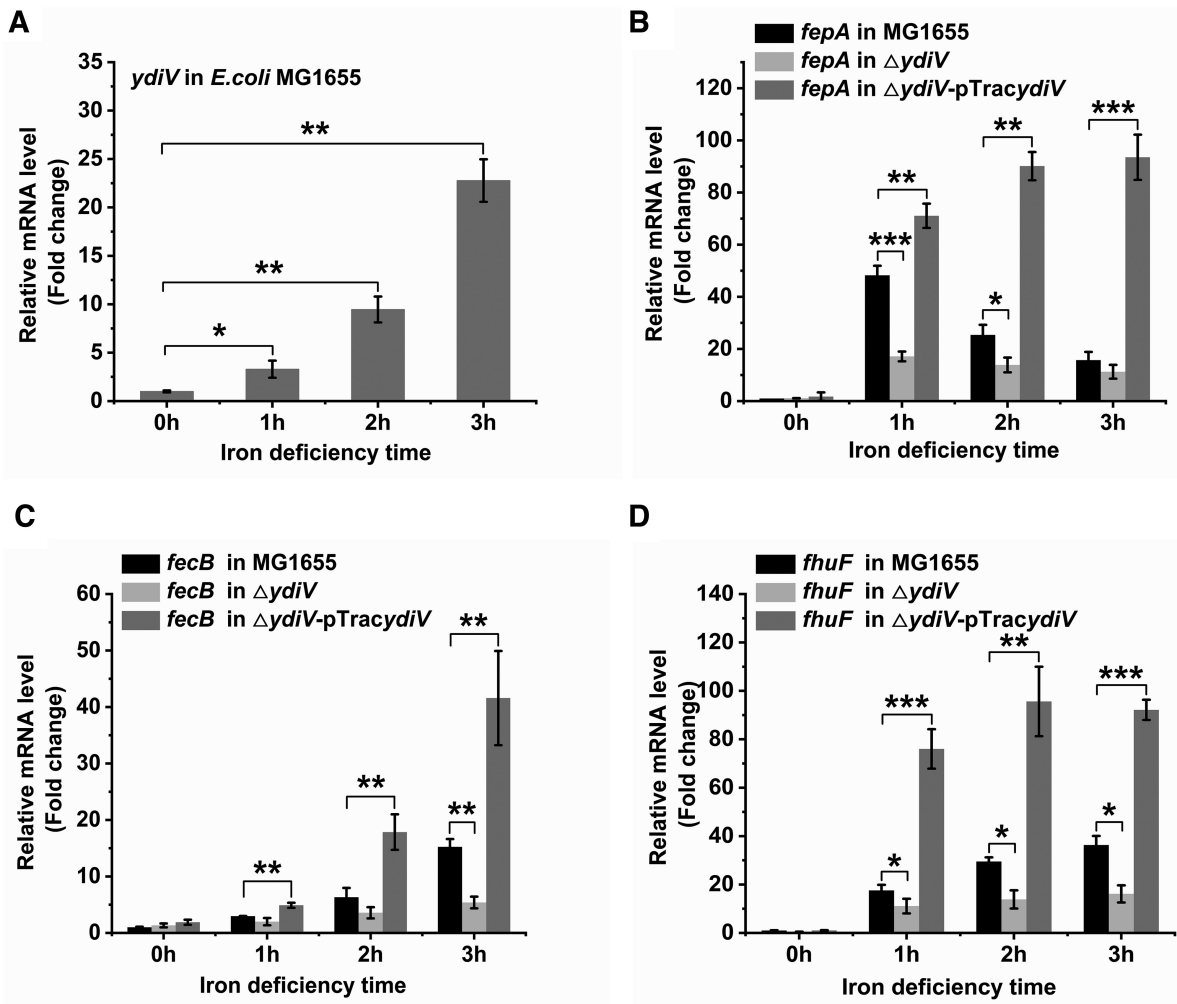
YdiV participates in *E. coli* MG1655 iron absorption. (**A**) The expression of *ydiV* in *E. coli* during iron starvation. Wild-type *E. coli* MG1655 was cultured in LB medium to an OD_600_ of 0.6, and then induced by 200 μM of 2,2′-dipyridyl. Samples were collected at 0–3 h after induction. The amount of *ydiV* mRNA was detected by qRT-PCR. The expression of *ydiV* under iron deficiency was compared with the expression of *ydiV* before iron deficiency (0 h) using the *t* test. (**B–D**) The expression of *fepA*, *fecB*, and *fhuF* genes in MG1655, Δ*ydiV*, and Δ*ydiV-*pTrac*ydiV* strains before (0 hours) and after iron-limitation was monitored by qRT-PCR. Strains were cultured in LB medium to an OD_600_ of 0.6, and then 200 μM of 2,2′-dipyridyl and IPTG were added to induce iron deficiency. Statistical significance is indicated as compared with MG1655 at the same time using a *t* test. All of the values shown represent the mean ± standard deviation of the results from three independent experiments. *P < 0.05; **P < 0.01; ***P < 0.001.

To test the association of *ydiV* with iron homeostasis, the expression of *ydiV* in *E. coli* K-12 MG1655 under iron-sufficient or limited conditions was monitored by qRT-PCR. No significant change was detected in *ydiV* expression in iron-sufficient LB medium (Supplementary Figure S2A). However, under iron deficient conditions, the expression of *ydiV* increased 3.3, 9.5 and 22.8-fold after 200 μM of 2,2′-dipyridyl was added for 1, 2 and 3 h, respectively (Figure 1A).

*Escherichia coli* has a complex signaling system to maintain iron homeostasis. When intracellular iron is low, bacteria sequentially induce the expression of iron-uptake genes to promote iron absorption. These genes include ferric Ent outer membrane transporter (*fepA*), ferric citrate ABC transporter periplasmic binding protein (*fecB*), and hydroxamate siderophore iron reductase (*fhuF*) (60,61). To verify whether YdiV plays a critical role in iron metabolism, the expression of *fepA*, *fecB*, and *fhuF* in wild-type *E. coli* MG1655, Δ*ydiV* and Δ*ydiV-*pTrac*ydiV* strains grown under iron-sufficient or iron-deficient conditions was monitored by qRT-PCR. When iron was sufficient, all of the iron-uptake genes were repressed—only *fhuF* in the Δ*ydiV-*pTrac*ydiV* strain showed a slight upregulation in transcription (Supplementary Figure S2B–D). However, under iron-deficient conditions, the regulation of these genes was highly dependent on YdiV (Figure 1B–D). Compared with wild-type *E. coli*, transcription of these genes in the Δ*ydiV-*pTrac*ydiV* strain showed a drastic increase (6.2-, 2.7-, and 2.6-fold increase for *fepA*, *fecB*, and *fhuF* at 3 hours, respectively) after 2,2′-dipyridyl treatment. By contrast, in the Δ*ydiV* strain, the upregulation of these genes was weaker than in wild-type *E. coli* (0.7-, 0.3- and 0.4-fold increase for *fepA*, *fecB*, and *fhuF* at 3 hours, respectively) (Figure 1B–D). These data suggest that the upregulation of *ydiV* is indeed a response to iron starvation and it promotes the activation of iron-uptake genes in *E. coli*.

### YdiV promotes the absorption of iron by regulating the DNA-binding ability of Fur

Since Fur is an overarching iron-responsive regulator, it is reasonable that YdiV performs its function through crosstalk with Fur. To clarify this assumption, we first confirmed that Fur was working properly in our *E. coli* strain. Transcription of the iron uptake genes (*fepA*, *fecB* and *fhuF*) was monitored in the wild-type and Δ*fur* strain. As expected, for the wild-type strain in LB medium, all three genes were strongly repressed by Fur (Supplementary Figure S3A). We then monitored the iron-dependent regulation of *ydiV* expression in the Δ*fur* strain by qRT-PCR. As expected, the mutation of *fur* did not influence the iron-dependent *ydiV* upregulation (Supplementary Figure S3B). The Δ*fur* strain, however, showed a very weak upregulation in the expression of iron uptake genes under iron-limited conditions (Supplementary Figure S3C–D). This suggests that YdiV is upstream of Fur and likely performs its function through Fur.

**Figure 2.**
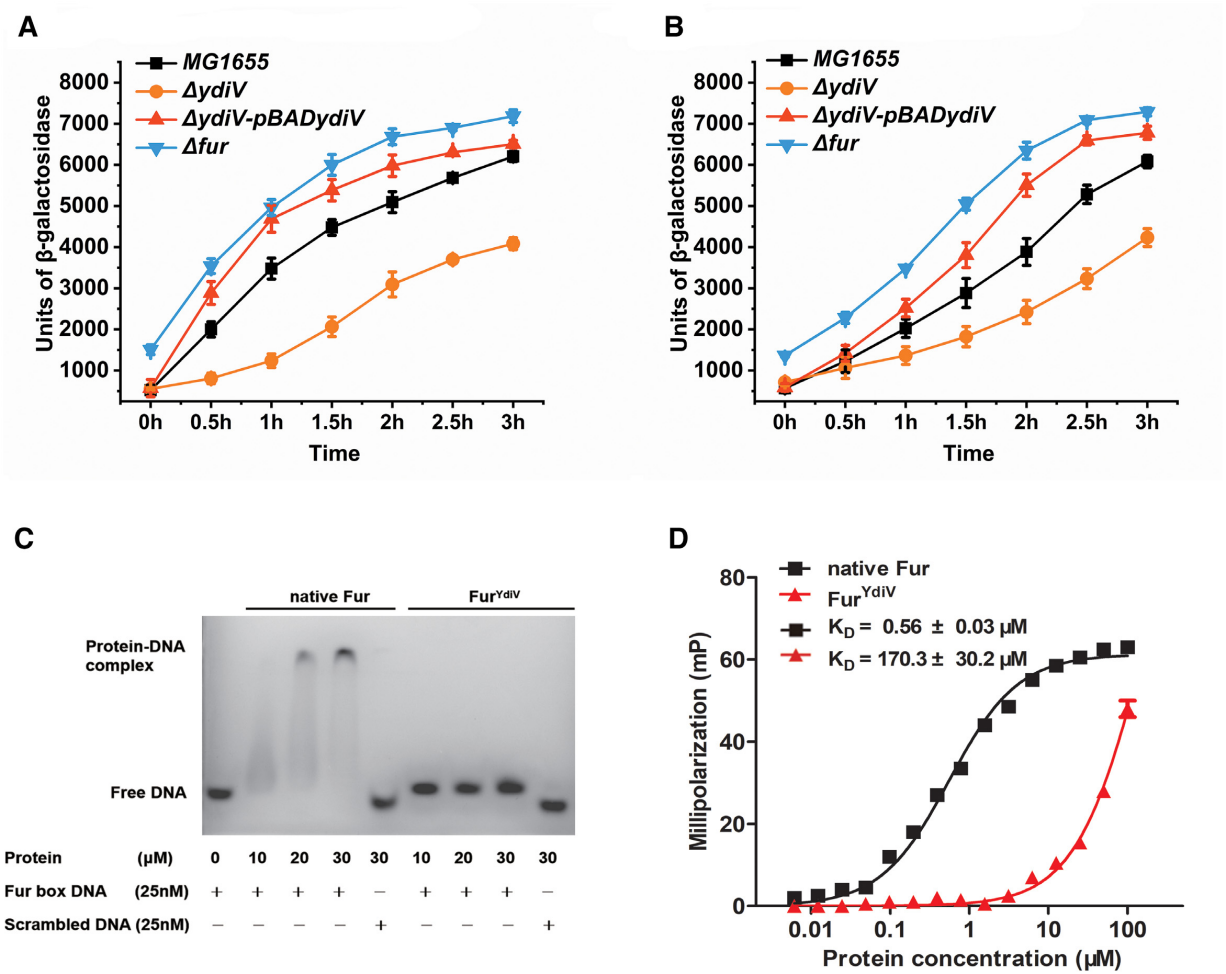
YdiV affects the DNA-binding ability of Fur. (**A, B**) The *fepA* promoter and *fhuF* promoter activities were tested in MG1655, Δ*ydiV*, Δ*ydiV-*pBAD*ydiV*, and Δ*fur* strains by β-galactosidase assays. The target strain was inoculated into LB medium and cultured to an OD_600_ of 0.2. Then 0.8 mg/ml arabinose and 200 μM of 2,2′-dipyridyl was added to reduce the available iron for the cells. Cultures at different induction times were collected to detect β-galactosidase activity. All of the values shown represent the mean ± standard deviation of the results from three independent experiments. (**C**) Electrophoretic mobility shift assay (EMSAs) for native Fur and Fur^YdiV^ with Fur box DNA. The FAM-labeled Fur box DNA or scrambled DNA (25 nM) was incubated with increasing amounts of native Fur or Fur^YdiV^ protein (the concentrations are noted in the panel) for 15 min at 37°C. The reaction buffer contained 100 μM MnCl_2_. The experiment was repeated three times with similar results, and a representative image is shown. (**D**) Fluorescence polarization analyses of native Fur and Fur^YdiV^ binding to Fur box DNA. FAM-labeled Fur box DNA (1 nM) was incubated with increasing amounts of native Fur or Fur^YdiV^ protein for 15 min at 37°C. The reaction buffer contained 150 μM MnCl_2_. The values shown are the mean ± standard deviation of three repeats.

To further clarify the influence of YdiV on Fur, a reporter plasmid containing the *fepA* or *fhuF* promoter and *lacZ* operon fusion (pCL *fepAp-lacZ* and pCL *fhuFp-lacZ*) was constructed. β-gal activity was then measured in different *E. coli* strains (MG1655, Δ*ydiV*, Δ*ydiV-*pBAD*ydiV* and Δ*fur*) under different iron conditions in order to evaluate Fur repression. These two promoters showed analogous results. During iron starvation, the Δ*fur* strain showed a higher β-gal activity as compared to the wild-type strain, demonstrating de-repression of the *fepA* and *fhuF* promoter. In the Δ*ydiV* strain, however, only a basal level of β-gal activity was observed, indicating that Fur was activated during iron-starvation. As expected, the *ydiV* complemented strain (Δ*ydiV*-pBAD*ydiV*) exhibited higher β-gal activity than the wild-type strain, demonstrating the de-repression of the *fepA* and *fhuF* promoter (Figure 2A and B). Under iron-sufficient conditions, similar results were also detected, but the activation of *fepA* and *fhuF* were weaker than that under iron-deficient treatment, and the *ydiV* complemented strain exhibited similar β-gal activity than the wild-type strain (Supplementary Figure S4). These data strongly suggested that YdiV specifically relieves Fur's repression on iron uptake.

qRT-PCR was used to test whether YdiV had an effect on Fur transcription. The results clearly showed that the mRNA level of *fur* did not significantly change along with the expression of YdiV during iron deficiency (Supplementary Figure S5). Thus, the regulation of Fur may occur after transcription.

In order to further determine the interaction between YdiV and Fur, pull-down assays were performed with co-expressed untagged YdiV and N-terminal His-tagged Fur protein in *E. coli* BL21 strain. However, we were never able to observe the YdiV-Fur complex. Only Fur was purified (Fur^YdiV^ protein) (Supplementary Figure S6). We then purified the native Fur protein (Fur protein overexpressed without YdiV) (Supplementary Figure S6) and performed a series of electrophoretic mobility shift assays (EMSAs) and fluorescence polarization (FP) experiments with Fur and Fur^YdiV^ to test whether YdiV had any effect on the DNA-binding ability of Fur. Surprisingly, while the native Fur readily bound to the Fur box DNA (51), the co-expressed Fur^YdiV^ protein did not form a complex with the Fur box DNA (Figure 2C). FP results showed that the binding affinity of Fur^YdiV^ for the Fur box DNA was about 300 times lower than that of native Fur (Figure 2D).

### YdiV changes the conformation of Fur

The four regulatory modes of Fur have been identified: apo-Fur activation, apo-Fur repression, holo-Fur activation, and holo-Fur repression (28,60,62). According to crystal structure of Fur in *Pseudomonas aeruginosa* and *Vibrio cholera*, each monomer of the dimeric Fur protein has two metal-binding sites—one is responsible for regulation and the other for structural stabilization (63,64). *Escherichia coli* Fur can bind two Zn^2+^ in each monomer (Zn_2_Fur), of which one is easily removed by treatment with zinc chelating agents, which leads to Zn_1_Fur. The remaining one can only be removed under denaturing conditions leading to apo-Fur (52). In *E. coli*, transcription of ferric uptake genes is regulated by holo-Fur repression: dimeric Fur binds to metal cofactors and combines with its target DNA operators repressing target genes transcription. Thus, metal cofactors and dimerization are critical for Fur function. In this part, we examined if these two important aspects of Fur are regulated by YdiV.

**Figure 3.**
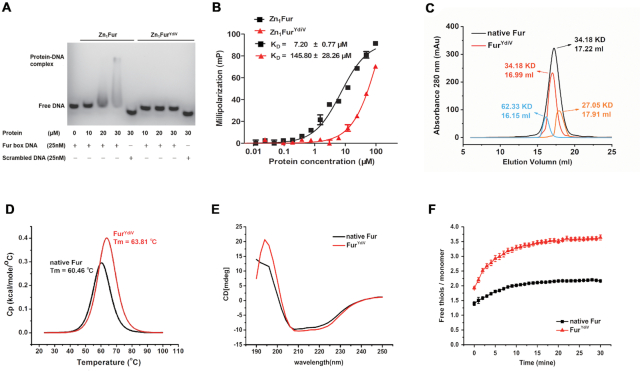
Comparison of native Fur and Fur^YdiV^ protein. (**A**) Electrophoretic mobility shift assay (EMSAs) for Zn_1_Fur and Zn_1_Fur^YdiV^ with Fur box DNA. The FAM-labeled Fur box DNA or scrambled DNA (25 nM) was incubated with increasing amounts of Zn_1_Fur and Zn_1_Fur^YdiV^ protein (the concentrations are noted in the panel). The experiment was repeated three times with similar results, and a representative image is shown. (**B**) Fluorescence polarization analyses of Zn_1_Fur and Zn_1_Fur^YdiV^ binding to Fur box DNA. FAM-labeled Fur box DNA (1 nM) was incubated with increasing amounts of Zn_1_Fur or Zn_1_Fur^YdiV^ protein. The values shown are the mean ± standard deviation of three repeats. (**C**) Size-exclusion chromatography results of native Fur and Fur^YdiV^ protein. The 62 kDa (blue) and 27 kDa (orange) proteins were used as markers for judging the oligomerization state of Fur (34.18 kDa). The molecular weight and elution volume of every peak is marked in the corresponding colors. (**D**) The *T*_m_ values of native Fur and Fur^YdiV^ were detected by VP-DSC and marked in the corresponding colors. The concentration of native Fur and Fur^YdiV^ was 100 μM. (**E**) Comparison of secondary structures of native Fur and Fur^YdiV^ protein. Far-UV CD spectra (190–250 nm) were obtained for native Fur and Fur^YdiV^ protein, which were diluted to 50 μg on a Jasco J-810 spectropolarimeter at 25°C. (**F**) The numbers of sulfhydryls in native Fur and Fur^YdiV^ were detected using Ellman's reagent, and the 412 nm absorbance value was scanning on the BioTek Synergy HT microplate detection system.

ICP-MS was performed to measure the metal content of Fur and Fur^YdiV^. The results showed that both forms of Fur contain the same amount of iron: each monomer contains one molecule of zinc and 0.8 molecules of iron. Then both Fur and Fur^YdiV^ were treated with EDTA to produce Zn_1_Fur and Zn_1_Fur^YdiV^ (52). The affinity of Zn_1_Fur and Zn_1_Fur^YdiV^ for Zn^2+^, Fe^2+^ and Fur box DNA was studied using ITC, EMSAs, and FP. Although these two proteins have different DNA affinities (7.2 μM for Zn_1_Fur and 145.8 μM for Zn_1_Fur^YdiV^) (Figure 3A–B), no significant difference were found in their affinity for metal ions (Supplementary Figure S7). Similarly, size-exclusion chromatography suggested both Fur and Fur^YdiV^ are dimeric proteins (Figure 3C). The slight difference in the elution volume implied a potential conformational difference between Fur and Fur^YdiV^. Indeed, a VP-DSC MicroCalorimeter indicated that Fur^YdiV^ has a higher thermal stability (*T*_m_ = 63.81°C) than Fur (*T*_m_ = 60.46°C) (Figure 3D). These data clearly showed that YdiV may perform its function by changing the structure or conformation of Fur.

CD and high-resolution HPLC–MS/MS were then performed to characterize Fur and Fur^YdiV^ in order to detect any other difference. Fur and Fur^YdiV^ exhibited almost the same CD spectrum, which suggested that these two forms of Fur have the same secondary structure composition and were not likely to have a global structural difference (Figure 3E). Unexpectedly, the high-resolution HPLC–MS/MS results showed that their molecular weights were different: 17 089 Da for Fur and 17091 Da for Fur^YdiV^. The two-dalton difference excludes the post-translation modification caused by YdiV, but rather suggests that Fur loses two hydrogen atoms while Fur^YdiV^ does not. Many Fur proteins from different sources contain four cysteines that exist in two Cys–XX–Cys motifs. One is equivalent to the Cys-93–XX–Cys-96 in *E. coli* Fur, the other motif occurs at the C-terminus immediately after the final beta-strand of the dimerization domain: Cys-133–XXXX–Cys-138. In the structure of *V. cholera* Fur, the first and third cysteines form a disulfide bond (64). In order to verify if a disulfide bond also forms in *E. coli* Fur, DTNB was used for a quick measurement of protein sulfhydryls (56). As expected, two reduced cysteines were detected in native Fur and four reduced cysteines detected in Fur^YdiV^ (Figure 3F). This suggested a disulfide bond in the native Fur protein is reduced when the intracellular YdiV concentration is high. This result further confirms that YdiV performs its function by changing the structure of Fur.

### Regulation of Fur by YdiV is SlyD-dependent

YdiV prevents Fur from binding to DNA *in vivo*, and thus, activates the iron absorption system of *E. coli*. To test if YdiV does the same *in vitro*, individually purified YdiV and native Fur were incubated together to determine if the DNA-binding activity of Fur was weakened. YdiV did not exert any effect on Fur activation *in vitro* even though the molar ratio of YdiV: Fur was as high as 10:1 (Supplementary Figure S8). This result strongly suggested that YdiV does not perform its function alone. Other intracellular proteins, most probably chaperones, are most likely involved in this process.

To identify which protein mediates the function of YdiV, a His-tagged Fur pull-down assay was performed. In order to reduce non-specific protein interference, the assay was improved with a single-step protease cleavage elution from the nickel column. Size-exclusion chromatography purification was then performed, followed by mass spectrometry (54). In total, 16 proteins were detected (Supplementary Table S6), including Fur, YdiV, FlhD and SlyD, which further verified the authenticity of the interaction between YdiV and Fur *in vivo*. The appearance of FlhD on the list was not surprising since it forms a stable complex with YdiV. The interaction of YdiV and FlhD leads to the formation of YdiV-FlhD_4_FlhC_2_, which then disassociates from the target DNA of FlhD_4_C_2_. This promotes the degradation of FlhD_4_FlhC_2_ by ClpXP protease, thereby inhibiting flagellar synthesis and bacterial mobility (38–41). In contrast, identification of SlyD was striking since it is a peptidyl-prolyl cis/trans isomerase and chaperone that facilitates protein folding. *E. coli* SlyD contains a PPIase FK506-binding protein (FKBP) domain and an insert-in-flap (IF) chaperone domain (65). However, SlyD has never been reported to associate with Fur in regulating iron homeostasis. Generally, SlyD is viewed as a common miscellaneous protein observed on nickel columns (66). Thus, further experiments were needed in order to verify the interaction.

**Figure 4.**
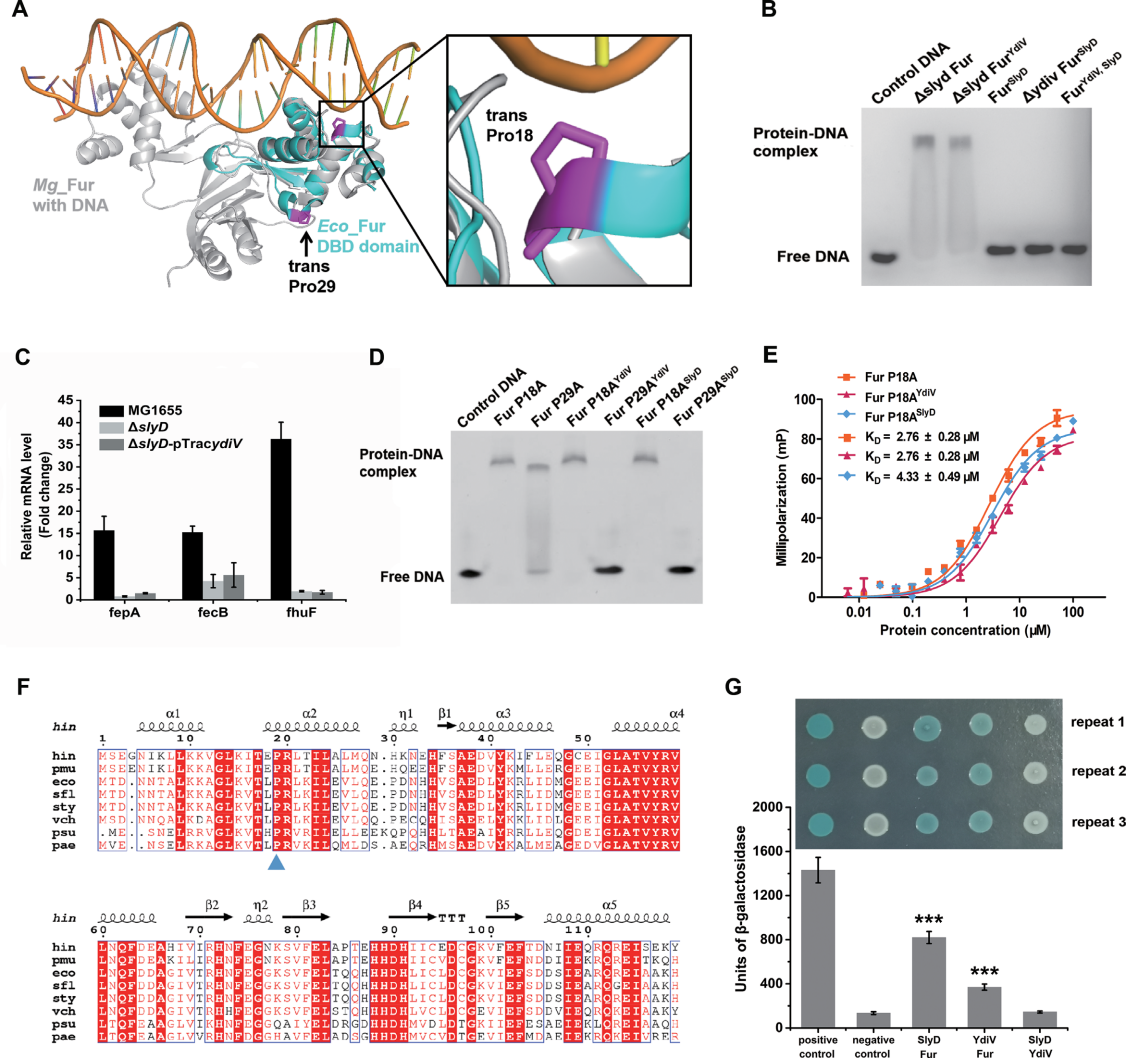
SlyD participates in the regulation of Fur by YdiV. (**A**) Structural comparison of *E. coli* Fur (*Eco*_Fur) DBD domain and *Magnetospirillum gryphiswaldense* Fur (*Mg*_Fur) with DNA complex. The two structures are superimposed and shown in cartoon mode (*Eco*_Fur: green, *Mg*_Fur: silver grey). The two trans prolines in the *Eco*_Fur DBD domain are indicated by arrows (purple). (**B**) EMSA of Fur purified from different strains with the Fur box DNA. The protein (30 μM) was mixed with 25 nM of DNA and placed at 37°C for 15 minutes before performing the EMSA. The reaction buffer contained 100 μM MnCl_2_. The experiment was repeated three times. (**C**) YdiV does not active expression of iron-uptake systems in the Δ*slyD* strain. The expression of *fepA*, *fecB*, and *fhuF* genes in MG1655, Δ*slyD*, and Δ*slyD-*pTrac*ydiV* strains during the induction of iron deficiency (3 h) was monitored by qRT-PCR. In order to induce iron limitation, 200 μM of 2,2′-dipyridyl was added to the LB medium. Three biological replicates were performed. (**D**) EMSA of Fur mutants purified from different strains with Fur box DNA. The experimental method is the same as Figure 4B. (**E**) Fluorescence polarization analyses of Fur P18A, Fur P18A^YdiV^, and Fur P18A^SlyD^ binding to Fur box DNA. FAM-labeled Fur box DNA (1 nM) was incubated with increasing amounts of different proteins. The reaction buffer contained 100 μM MnCl_2_. The values shown are the mean ± standard deviation of three repeats. (**F**) CLUSTALW alignment between *E. coli* Fur and Fur from other pathogenic bacteria. One hundred and twenty residues (1–120 aa) of Fur were used in this alignment, and the most conserved proline residue is marked by triangle. hin: *Haemophilus influenzae*; pmu: *Pasteurella multocida*; eco: *Escherichia coli*; sfl: *Shigella flexneri*; sty: *Salmonella enterica*; vch: *Vibrio cholerae*; psu: *Pseudoxanthomonas suwonensis*; and pae: *Pseudomonas aeruginosa*. (**G**) Direct interaction between YdiV, Fur, and SlyD was detected by a bacterial two-hybrid method. Positive control: recombinant strain BTH101 containing T18-zip and T25-zip vectors. Negative control: recombinant strain BTH101 containing the empty T18 and T25 vectors. Corresponding strains were tested by β-galactosidase assays and LB–X-Gal plates, which displayed above the histogram. The values represent the mean ± standard deviation of three repeated results and statistical significance is indicated by ***P < 0.001 as compared with negative control using a *t* test.

All Fur orthologues contain an N-terminal DNA-binding domain (DBD domain) and a C-terminal dimerization domain. The DBD domain contains a winged helix motif, and up to four of these motifs can bind a 19-bp inverted repeat sequence known as a Fur-box (67). The N-terminal part of *E. coli* Fur contains two trans prolines: Pro18 and Pro29. Structural comparison of *E. coli* Fur DBD domain and *Magnetospirillum gryphiswaldense* Fur-DNA complex shows that these two prolines (Pro18 and Pro29) are located on the opposite ends of the second α-helix of DBD domain, implying their potential roles in DNA binding (Figure 4A) (68,69). We speculated that cis/trans isomerization of these two prolines would cause a conformational change in the DBD domain, and thus, would alter its DNA-binding ability. Therefore, it is reasonable that Fur is a novel substrate of SlyD, which connects SlyD and iron homeostasis.

To confirm the involvement of SlyD in iron homeostasis, we constructed the BL21 Δ*slyD* strain and used it to express Fur (Δ*slyD* Fur) and Fur^YdiV^ (Δ*slyD* Fur^YdiV^) (Supplementary Figure S6). The DNA-binding ability and sulfhydryls content of these two proteins were then tested using an EMSA and DTNB. Interestingly, Δ*slyD* Fur and Δ*slyD* Fur^YdiV^ all retained their DNA-binding ability (Figure 4B) and formed a disulfide bond (containing about two sulfhydryls) (Table 1), indicating that YdiV-dependent regulation of Fur is SlyD-dependent. In order to test if SlyD is also dependent on YdiV, we constructed the BL21 Δ*ydiV* strain and co-expressed untagged SlyD and His-tagged Fur in wild-type BL21 (Fur^SlyD^), BL21 Δ*ydiV* strain (Δ*ydiV* Fur^SlyD^) and YdiV overexpression BL21 strain (Fur^ydiv, SlyD^) (Supplementary Figure S6) to evaluate their DNA-binding ability and sulfhydryl content. According to our results, all three proteins did not bind to DNA (Figure 4B) and had no disulfide bond (contained about four sulfhydryls) (Table 1). This indicates that when overexpressed inside cells, SlyD itself can disable Fur's ability to bind DNA. It is worth noting that the DNA-binding ability of different Fur proteins was positively related to their disulfide bond formation. This implies that YdiV and SlyD regulate the DNA-binding ability of Fur by changing its conformation.

**Table 1.** The sulfhydryl content of different Fur proteins. The sulfhydryl content was quickly measured via Ellman's reagent (DTNB). The values shown are the mean ± standard deviation of three repeats

Protein	Sulfhydryls	Protein	Sulfhydryls
Δ*slyD* Fur	1.94 ± 0.11	Fur^YdiV, SlyD^	3.95 ± 0.12
Δ*slyD* Fur^YdiV^	1.93 ± 0.09	Fur P18A	1.97 ± 0.11
Fur^SlyD^	3.99 ± 0.13	Fur P18A^YdiV^	2.03 ± 0.15
Δ*ydiV* Fur^SlyD^	3.92 ± 0.15	Fur P18A^SlyD^	1.99 ± 0.07

To further confirm that SlyD is involved in iron homeostasis *in vivo*, Δ*slyD* and Δ*slyD-*pTrac*ydiV* strains of *E. coli* MG1655 were constructed and their response to iron starvation was tested using qRT-PCR. As expected, the response of these strains to iron starvation was largely weakened as compared to wild-type MG1655, and overexpression of *ydiV* gene did not upregulate the expression of iron acquisition genes without *slyD* (Figure 4C). Under iron sufficiency, all of the iron-uptake genes were repressed in all strains, including MG1655, Δ*slyD*, or Δ*slyD-*pTrac*ydiV* strains (Supplementary Figure S9).

The involvement of SlyD in iron homeostasis raises a question of whether its function is dependent on peptidyl-prolyl cis-trans isomerase activity. Since Fur contains two proline residues (P18 and P29) in its DBD, site-directed mutagenesis was employed to identify the proline residue on which the isomerization occurs. Two mutants of Fur (P18A, P29A) were constructed and purified from *E. coli* BL21 Δ*fur* strain and BL21 Δ*fur* overexpressing YdiV or SlyD strain, and their ability to bind Fur box DNA was investigated using EMSA assays. When purified in the BL21 Δ*fur* strain, both Fur mutants P18A and P29A retained the ability to bind to DNA. When purified from the BL21 Δ*fur* overexpressing YdiV or SlyD strain, however, these two mutants showed quite different behaviors. Fur P29A lost its DNA-binding ability, but Fur P18A retained its DNA-binding ability, indicating that Pro18 is the target of SlyD (Figure 4D). The DNA affinity of Fur P18A, Fur P18A^YdiV^, and Fur P18A^SlyD^ were compared using FP measurements. From the results, these three proteins had similar DNA affinities, which was about 6–8 times lower than native Fur (Figure 4E). We then used DTNB to measure the sulfhydryl content of Fur P18A, Fur P18A^YdiV^ and Fur P18A^SlyD^ proteins. Our results showed that all three proteins formed a disulfide bond which contained about two sulfhydryls) (Table 1). This confirmed that YdiV and SlyD changed the conformation of Fur through the cis-trans isomerization of Pro18, which caused Fur to lose its DNA-binding ability. We believe that proline isomerization may not directly affect the redox state of Fur cysteines, but the resulting conformational change may cause spatial separation of them, thus preventing the formation of disulfide bond. Sequence alignment showed that Pro18 is highly conserved in Fur orthologs from many pathogenic bacteria, suggesting that this mechanism could exist across different species (Figure 4F).

After confirming the iron regulation function of YdiV, the flagellar synthesis function of YdiV (38–40) was also detected in WT, Δ*fur*, and Δ*slyD* strains. According to our results, knockout and overexpression of *ydiV* have similar effects on mobility in different strains (Supplementary Figure S10A and B). Thus, the role of YdiV in iron acquisition is separate from its essential role in flagellar biosynthesis, which is crucial for *E. coli* infection.

### YdiV and SlyD work cooperatively to regulate Fur

Our results have confirmed the involvement of YdiV and SlyD in iron homeostasis and have shown that YdiV is dependent on SlyD; however, high levels of SlyD can regulate Fur without YdiV. To determine whether SlyD can perform this function independently, we tested the transcription of *slyD* in wild-type *E. coli* MG1655, Δ*ydiV*, and Δ*ydiV-*pTrac*ydiV* strains during iron starvation using qRT-PCR. Significantly, the transcription of *slyD* did not increase, instead it decreased slightly during iron deficiency (Supplementary Figure S11). These results were consistent with the fact that *slyD* has been used as a housekeeping gene in many assays for its stable expression under six different conditions (exponential phase, cold shock, oxidative and cold shock combined, physiological water, oxidative stress, and stationary phase) (70). This strongly suggested that SlyD does not respond to iron starvation directly, rather it mediates the response of YdiV to iron starvation.

The regulation of Fur by YdiV and SlyD implis an interaction between these three proteins. The process of identifying SlyD also indicated that YdiV, SlyD and Fur may form a complex. However, neither a tertiary complex nor a binary complex was ever purified. Thus, we speculated that Fur only forms a transient complex with YdiV or SlyD during folding and once the folding process is over, the transient complex dissociates immediately. To test this hypothesis, we constructed a bacterial two-hybrid system based on adenylate cyclase reconstitution (57). The strong interaction between T18-zip and T25-zip plasmids was used as positive controls and the empty T18 and T25 plasmids as negative controls. The interaction of Fur and SlyD was detected to be six times as strong as the negative control. The interaction of Fur and YdiV was three times as strong as the negative control (Figure 4G). However, an interaction between SlyD and YdiV was not observed. These results suggested that SlyD catalyzes the isomerization of Fur, and YdiV facilitates this process by binding to SlyD-bound Fur.

### Fur and Fur^YdiV,SlyD^ have different conformations

**Figure 5.**
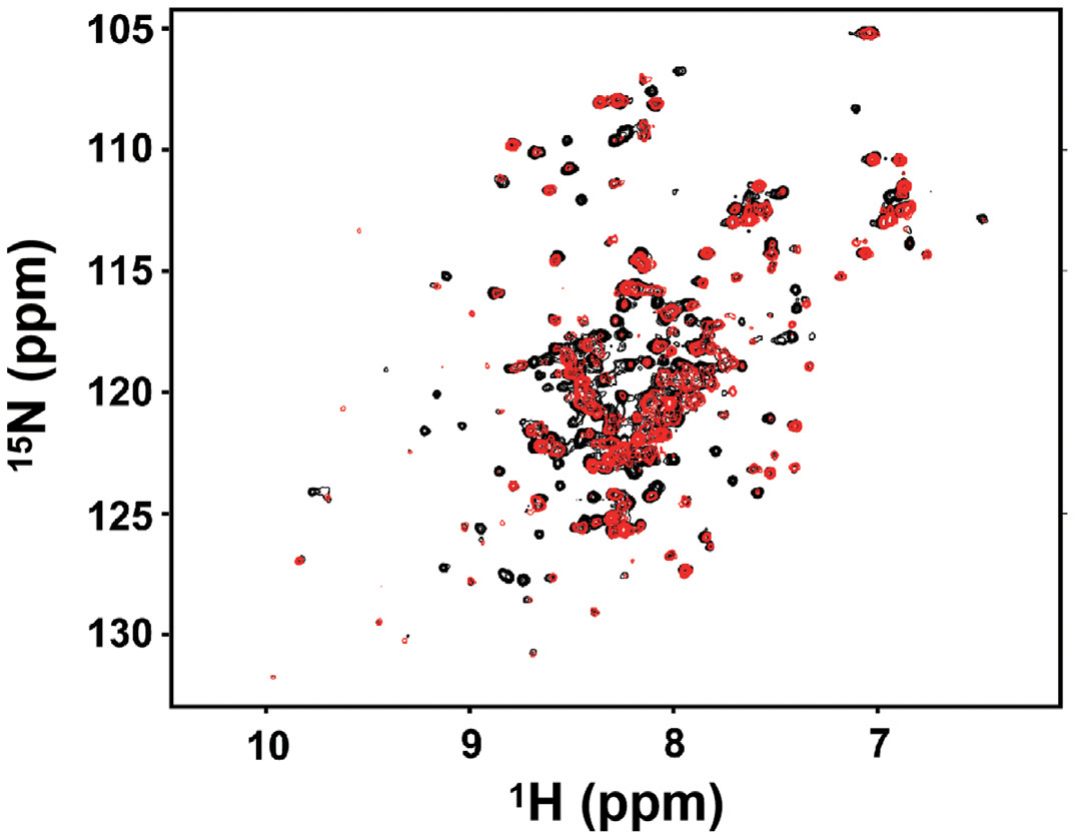
Comparison of the NMR spectra of native Fur and Fur^YdiV,SlyD^. The ^1^H and ^15^N chemical shift signals of native Fur (black) and Fur^YdiV,SlyD^ (red) were detected by HSQC experiments and analyzed by the NMRPipe software.

Thus far, our data have been consistent with YdiV-facilitated and SlyD-catalyzed isomerization of Fur during the protein-folding process. To test if this results in different conformations of Fur, we attempted to crystalize Fur^YdiV,SlyD^. However, we were not successful in crystalizing Fur^YdiV,SlyD^. Thus, in order to obtain information on the structural differences, Fur and Fur^YdiV,SlyD^ were characterized using 2D ^1^H–^15^N HSQC experiments. Our results clearly showed that although most of the chemical shift peaks of Fur and Fur^YdiV,SlyD^ overlapped, many were different. This was consistent with our hypothesis that Fur and Fur^YdiV,SlyD^ are similar in overall structure, but they differ in some local regions, which may affect DNA binding (Figure 5).

### Comparison of the inhibitory activities of Fur in different forms

**Figure 6.**
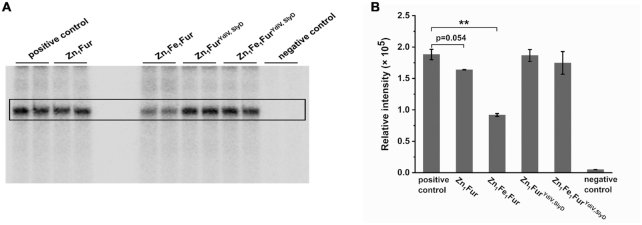
The *in vitro* transcription inhibitory activities of Zn_1_Fe_1_Fur, Zn_1_Fur, Zn_1_Fe_1_Fur^YdiV, SlyD^ and Zn_1_Fur^YdiV, SlyD^. The concentration for all Fur proteins was 500 nM. Positive control: transcription system without Fur in any form. Negative control: transcription system without DNA template. (**A**) Radiolabeled RNA products during *in vitro* transcription. (**B**) The corresponding band densitometry quantified from the results of RNA production and shown as the mean ± standard deviation of two replicates. Statistical significance is indicated as compared with positive control using a *t* test. **P < 0.01.

According to the classic model of iron regulation, under iron-sufficient conditions the iron-binding Zn_1_Fe_1_Fur protein combines with the operator site of a target promoter, inhibiting the transcription of iron-uptake genes. Under iron-limited conditions, Fur releases the regulatory Fe^2+^, and Zn_1_Fur dissociates from the target DNA sequence, relieving the gene repression (21,22). In this experiment, we discovered that high intracellular levels of YdiV resulted in the formation of Fur^YdiV, SlyD^ protein. Fur^YdiV, SlyD^ lost the ability to bind to DNA, in the absence and presence of Fe^2+^, thus relieving the repression on iron-uptake genes. To examine which mechanism is more efficient, we purified Zn_1_Fe_1_Fur (native Fur), Zn_1_Fur (native Fur treated with EDTA), Zn_1_Fe_1_Fur^YdiV, SlyD^ (Fur^YdiV, SlyD^) and Zn_1_Fur^YdiV, SlyD^ (Fur^YdiV, SlyD^ treated with EDTA) separately, and tested their effects on transcription *in vitro*. Our results showed that Zn_1_Fe_1_Fur significantly inhibited transcription. Zn_1_Fur had a weakened inhibitory effect on transcription. Zn_1_Fe_1_Fur^YdiV, SlyD^ and Zn_1_Fur^YdiV, SlyD^, however, had almost completely lost their ability to repress transcription (Figure 6A and B). These data indicated that the YdiV-SlyD-Fur pathway is more efficient in responding to iron deficiency.

### YdiV and SlyD are essential for the survival and growth of UPEC inside BECs

UPEC is the causative agent of over 85% of recurrent urinary tract infections. Iron has been known to be the key factor for infection. UPEC secretes a number of siderophores to compete with the host for iron, which is always deficient, especially inside the host cells. In addition to siderophores, UPEC has evolved other countermeasures to deal with iron deficiency inside the host cells. These measures include exploitation of host Rab35 for iron acquisition and taking advantage of ferritinophagy of autophagosomal and lysosomal compartments for increasing iron capture (71,72).

**Figure 7.**
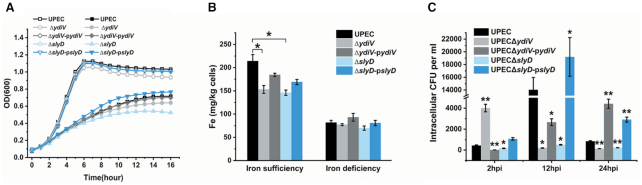
The effect of YdiV and SlyD on UPEC iron metabolism and pathogenicity. (**A**) The growth curves of UPEC and UPEC recombinant strains. The hollow symbols represent strains cultured in M9 medium with 20 μM of FeCl_3_. The solid symbols represent strains cultured in M9 medium with 200 μM of 2,2′-dipyridyl. The values represent the mean ± standard deviation of three repeated results. (**B**) The intracellular iron content of UPEC and UPEC recombinant strains was tested by ICP-MS. Iron sufficiency represents LB medium and iron deficiency represents LB medium with 200 μM of 2,2′-dipyridyl. The values represent the mean ± standard deviation of three repeated results. Statistical significance is indicated as compared with wild-type UPEC using a *t* test. *P < 0.05. (**C**) The intracellular growth of UPEC and *ydiV* or *slyD* mutant strains. The colony forming units (CFUs) of intracellular UPEC and UPEC recombinant strains were quantified in BECs at the indicated hours post-infection (hpi). All of the values shown represent the mean ± standard deviation from six independent experiments. Statistical significance is indicated as compared with wild-type UPEC at the same hpi using a *t* test. *P < 0.05; **P < 0.01.

In order to verify the effect of SlyD and YdiV on UPEC iron metabolism, we constructed UPEC Δ*ydiV*, UPEC Δ*slyD*, and their corresponding complementary strains to test their growth in M9 media with different iron conditions. As expected, in iron-limited conditions (M9 medium with 200 μM of 2,2′-dipyridyl), Δ*ydiV* and Δ*slyD* strains grew slower than wild-type UPEC. Complementation of *ydiV* or *slyD* strains restored the growth back to wild-type levels. In iron-rich conditions (M9 medium with 20 μM FeCl_3_), the strains showed similar growth curves (Figure 7A). We then measured their iron content using ICP-MS and found that mutations of *ydiV* and *slyD* caused a decrease in iron content, whether in iron-rich or iron-limited environments. Both complementary strains contained more iron than the corresponding mutant strains (Figure 7B).

Since acquisition of iron is a prerequisite to UPEC infection and YdiV and SlyD may cooperatively regulate iron acquisition of UPEC, we hypothesized that YdiV and SlyD could be essential for UPEC survival and growth inside host cells. To test our hypothesis, a bacterial invasion assay was performed to test the ability of wild-type UPEC, UPEC Δ*ydiV*, UPEC Δ*slyD*, and the corresponding complementary strains to invade and grow inside human bladder carcinoma cell line 5637. Intracellular CFU data at 2-hour post-infection (hpi) showed that the wild-type UPEC successfully invaded BECs. Rapid growth of wild-type UPEC was indicated by the significantly higher CFUs at 12 hpi. However, a sharp decrease in the CFUs at 24 hpi indicated that most UPEC were eliminated, although a certain number of bacteria still survived. Intriguingly, UPEC Δ*ydiV* had a much more successful invasion rate, as indicated by the CFUs at 2 hpi compared with the other strains. This could be due to the upregulation of the flagellar genes and bacteria motility. However, CFUs at 12 and 24 hpi suggested that UPEC Δ*ydiV* completely lost the ability to survive and grow inside cells. For UPEC Δ*slyD*, only trace amounts invaded and survived inside cells. UPEC strains with complementary expression of either YdiV or SlyD retained a much higher CFU at 24 hpi as compared to the wild-type strain. These data strongly suggested that YdiV and SlyD are essential for UPEC infection. UPEC Δ*ydiV* and UPEC Δ*ydiV-*p*ydiV* had a complex phenotype because YdiV not only regulated iron homeostasis, but also acted as a repressor of flagellar genes (Figure 7C).

## DISCUSSION

Since iron is both necessary and toxic to bacteria, the amount of iron inside bacterial cells must be tightly controlled. Since it was identified over 30 years ago in *E. coli*, Fur has been demonstrated to be the central regulator of iron homeostasis in numerous bacteria (73). Extensive studies led to a widely accepted model on Fur regulation in response to different iron conditions. According to this model, at high Fe^2+^ concentrations, Fe^2+^-loaded Fur binds to Fur box DNA upstream of the promoter of the regulated genes and represses the expression of iron acquisition genes. When the intracellular iron concentration is low, Fe^2+^ dissociates from Fur, which makes Fur lose DNA-binding capacity and de-represses iron acquisition genes (21,22).

This classic model relies on the feedback regulation of Fe^2+^-Fur binding. In recent years, however, scientists have shown some diverse results in the regulation of homologous Fur proteins. For instance, both the ion-bonding site 1 and site 2 mutants of *Bradyrhizobium japonicum* Fur were able to repress gene expression *in vivo* (74). The *Campylobacter jejuni* apo-Fur is able to dimerize and bind to its target promoter DNA sequence (28). These phenomena imply that there are other mechanisms regulating Fur protein function. In this study, we proposed a novel mechanism dependent upon the YdiV-SlyD-Fur axis, which does not directly depend on the binding of Fe^2+^. Our results complement the traditional holo-Fur regulation mechanism.

### Model for YdiV-Fur-SlyD-dependent iron metabolism

In this study, we found that the expression of the *ydiV* gene was upregulated in an iron-deficient environment, and high levels of YdiV transforms Fur into a novel form, which does not bind DNA in a SlyD-dependent manner. This form of Fur (Fur^YdiV, SlyD^) contains the same number of Zn^2+^ and Fe^2+^ ions as the active native Fur, but it has different conformation. Thus, at least in *E. coli*, Fur can sense the concentration of YdiV to maintain iron homeostasis, and the expression level of YdiV is associated with the intracellular iron concentration. Thus, our findings established a new model about how *E. coli* employs the Fur-YdiV-SlyD axis to maintain iron homeostasis under different iron conditions.

**Figure 8.**
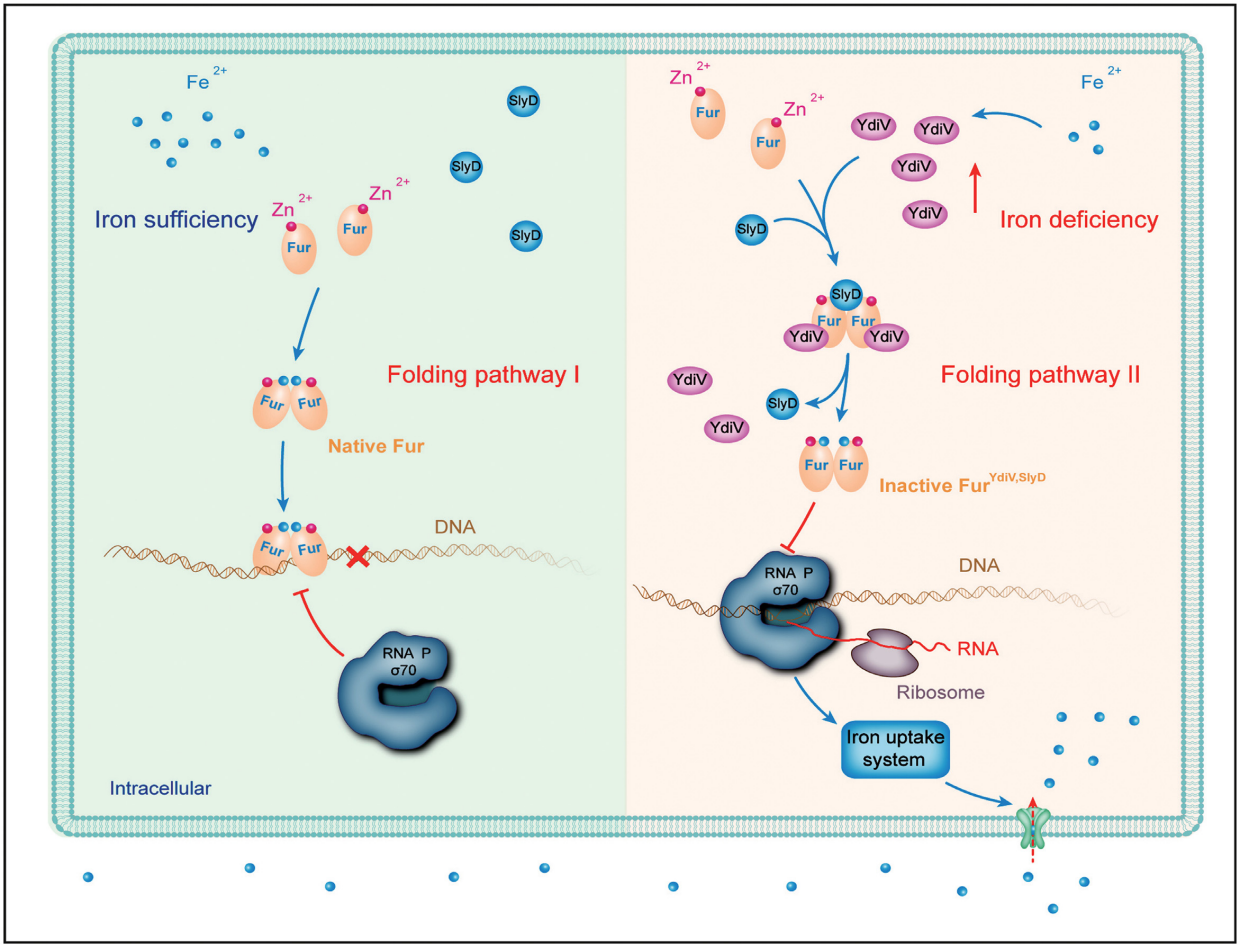
Model for YdiV-Fur-SlyD-dependent iron homeostasis. High levels of YdiV and SlyD convert Fur into a conformation that causes it to lose its DNA-binding ability, which, in turn, induces the gene expression of the iron-uptake systems.

In an iron-rich environment, the expression of YdiV is low and there is certain amount of SlyD inside the bacterial cell. Transcription and translation of *fur* gene are maintained at a steady rate. Most of the nascent Fur peptides fold into a dimer with the normal conformation containing a disulfide bond in each monomer (Folding pathway I, native Fur). Some of the remaining nascent peptides are recognized by SlyD, but without the help of YdiV, only a small fraction of them fold into the new conformation. Since normal Fur is dominant under this condition, most Fur box DNA is occupied by Fur, which occludes the RNA polymerase and represses the corresponding iron acquisition genes. When *E. coli* enters an iron-deficient environment, such as invasion of host cells, the expression of YdiV is stimulated by unknown mechanisms. A high concentration of YdiV promotes the formation of a transient tertiary YdiV-Fur-SlyD complex, which reduces the disulfide bonds in Fur and leads to a more stable Fur without DNA-binding ability (Folding pathway II, Fur^YdiV,SlyD^). Once Fur^YdiV,SlyD^ is produced, the transient tertiary complex dissociates releasing YdiV and SlyD to catalyze formation of more Fur^YdiV,SlyD^. Consequently Fur^YdiV,SlyD^ becomes dominant and most of the Fur box DNA is free, which de-represses iron acquisition genes (Figure 8).

The mechanism described above depends on the peptidyl-prolyl cis-trans isomerase activity of SlyD, which targets Pro18 of Fur. During this process, YdiV functions as an essential helper molecule of SlyD making isomerization reactions easier and faster. By far, we do not know if a *cis*-peptide bond exists in Fur^YdiV,SlyD^, or if isomerization only occurs during the folding process. Future research on the structure of Fur^YdiV,SlyD^ may answer this question.

### The advantage of the YdiV-Fur-SlyD axis

Nutritional immunity and pattern-recognition receptors (PRRs), triggered following immune reactions, pose deadly threats to intracellular pathogens. Strikingly, flagellin can be detected by the host PRR TLR5, which triggers pyroptosis of macrophages (75,76). To survive and grow inside the host cells, intracellular pathogens have developed complex signaling systems to counteract host immunity. Formation of the YdiV-Fur-SlyD axis is an effective mechanism to tightly coordinate the countermeasures of the bacteria, which enables UPEC to grow inside and outside of the host cells.

Outside of the host cells, where iron is relatively high (35), expression of YdiV is low and its inhibition on flagella is relieved. The expression of flagella confers motility on UPEC, which is beneficial to invasion (23,24). Once inside the host cells, UPEC encounters iron-starvation conditions (34), which upregulates YdiV expression. This not only represses flagella expression, which renders *E. coli* invisible to the host immune system (23,24), but it also induces iron acquisition systems, which helps *E. coli* overcome host nutritional immunity (3–5). As a result, UPEC successfully survives and grows within host cells. Without the YdiV-Fur-SlyD axis, although Fur itself can sense the intracellular iron concentration, its full function needs Zn_1_Fe_1_Fur convert to Zn_1_Fur. This process would need a sharp decline in the intracellular iron concentration, which is obviously disadvantageous for survival. Furthermore, the YdiV-Fur-SlyD axis is an efficient mechanism that effectively detects and responds to an iron-deficient environment before iron deficiency occurs within the bacterial cell (Figure 4C).

We do not know how iron deficiency triggers the upregulation of YdiV. It has been reported that autoinducer-1 stimulates *ydiV* expression in an *sdiA*-dependent manner (77). Strikingly, a *Pseudomonas* quinolone signal (PQS), which acts as an iron chelator, upregulates a number of genes for iron acquisition and the oxidative stress response (78,79). These studies imply the possible involvement of a quorum-sensing system in YdiV-Fur-SlyD-dependent iron regulation in *E. coli*. However, given the complexity of the iron regulation system and environment inside host cells, other unknown mechanisms may also exist. A recent study indicated that reactive oxygen species (ROS) can also trigger the overexpression of *ydiV*, which improved the ability of *E. coli* to resist oxidative stress; however, this mechanism was independent of SlyD (unpublished data). This emphasizes the importance and complexity of YdiV.

### SlyD is a chaperone which affects the final conformation of Fur

SlyD is a peptidyl-prolyl *cis*/*trans* isomerase (PPIase) and chaperone (65). Under anaerobic conditions *in vivo*, SlyD can specifically influence the balance of nickel ions in the cell (80) and serves as a Ni^2+^ reservoir for [NiFe]-hydrogenase biosynthesis (81). SlyD has been shown to interact directly with HypB, an accessory protein required for hydrogenase maturation (82), and transfers Ni^2+^ to HypB (81). SlyD also interacts directly with HycE, the large subunit of hydrogenase 3, via its IF domain (83). Moreover, SlyD is required for phage φX174-induced cell lysis by stabilization of the φX174 lysis protein E (84). In this experiment, we first demonstrated that SlyD is involved in the regulation of iron metabolism in a novel way.

In general, chaperones are proteins or protein complexes that facilitate the process of protein folding without affecting their final structure or conformation. It is surprising that SlyD not only facilitates protein folding, but it also switches the protein folding pathway, thus changing the final structure of the protein with the help of YdiV. Because YdiV and SlyD are highly conserved across enteric bacteria, this mechanism may also be present in most enteric bacteria. More than that, SlyD homologues widely exist in all prokaryotic and eukaryotic organisms. As such, this mechanism may exist in all organisms, although the helper molecules may differ (85,86).

### YdiV is a potential target for the development of antibacterial drugs and vaccines

UPEC is responsible for over 85% of recurrent urinary tract infections, which are generally difficult to cure due to the tolerance of UPEC to multi-antimicrobials (87). Iron has been known to be the key factor for UPEC infection; thus, iron acquisition systems are a good target for new antimicrobials. Since Fur is the central regulator of iron homeostasis, developing an inhibitor of Fur may provide this alternative. However, the fact that the *fur* mutant of UPEC has been demonstrated to be more virulent than the wild-type strain refutes this idea (32). In this respect, our finding that YdiV is dominantly involved in iron homeostasis makes YdiV a good target for drug development. Based on our studies, an inhibitor of YdiV will inhibit the expression of iron acquisition systems and also induce the expression of flagella. This subjects UPEC to iron-starvation within the host cells and also exposes UPEC to intracellular pattern recognition receptors (PPRs). In addition, YdiV also mediates resistance to oxidative stress (unpublished results). Thus, an inhibitor of YdiV could weaken three major mechanisms UPEC uses against the host innate immune system.

Moreover, UPEC Δ*ydiV* has an increased invasion rate as compared to the wild-type strain, but it fails to survive inside the host cells. This means that UPEC Δ*ydiV* could trigger strong immune reactions, but is safe to the host as it does not sustain infection. Thus, it has the potential to be used as a vaccine for susceptible populations.

## Supplementary Material

gkaa696_Supplemental_File
