# Supplementary material for: YdiV regulates Escherichia coli ferric uptake by manipulating the DNA-binding ability of Fur in a SlyD-dependent manner
Source: Nucleic Acids Res. 2020 Aug 19;48(17):9571–88. doi: 10.1093/nar/gkaa696 (PMC7515728; doi:10.1093/nar/gkaa696)
Supplement: gkaa696_Supplemental_File [file gkaa696_supplemental_file.pdf]

YdiV regulates *Escherichia coli* ferric uptake by manipulating the DNA-binding ability of Fur in a SlyD-dependent manner

SUPPLEMENTARY DATA

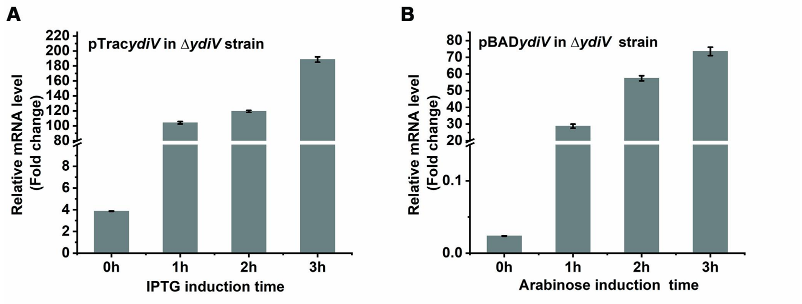

Supplementary Figure S1. Expression of *ydiV* from pTrac*ydiV* and pBAD*ydiV* plasmids increases over time after induction. (**A**) The Δ*ydiV* strain containing pTrac*ydiV* plasmid was cultured in LB medium and induced by 0.3 mM IPTG. (**B**) The Δ*ydiV* strain containing pBAD*ydiV* plasmid was cultured in LB medium and induced by 1 mg/mL L-Arabinose. The mRNA level of *ydiV* was tested by qRT-PCR and compared with *gapA* expression at the same point in time. All the values shown represent mean ± standard deviation from three independent experiments.

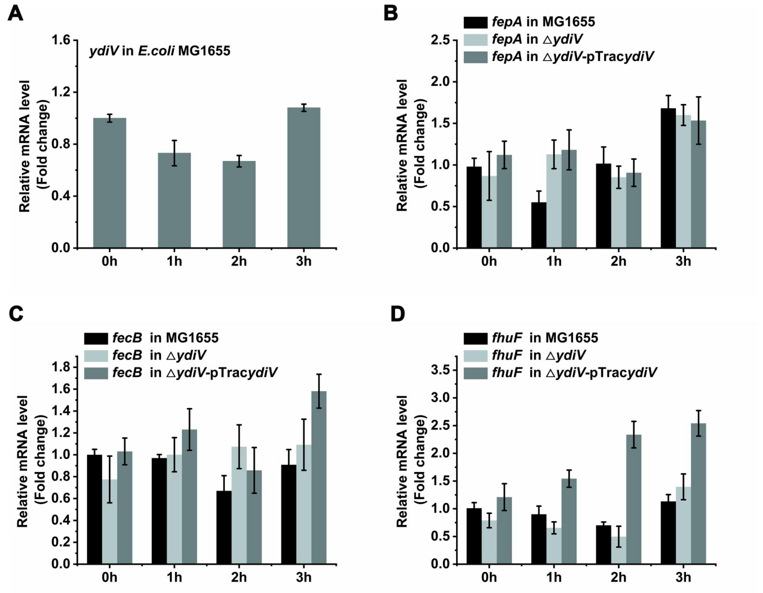

Supplementary Figure S2. *ydiV* as well as iron uptake genes show low expression under iron sufficient condition. (**A**) The expression of *ydiV* in *E. coli*. Wild-type *E.coli* MG1655 was cultured in LB medium to OD_{600}=0.6, then samples were collected at 0 to 3h synchronously with figure 1A. The amount of *ydiV* mRNA was detected by qRT-PCR and compared with 0h. (**B–D**) The iron uptake genes expression in MG1655, Δ*ydiV* and Δ*ydiV*-pTrac*ydiV* strains. Strains were cultured in LB medium to OD_{600}=0.6, then IPTG were added for *ydiV* induction.

Samples were collected at 0 to 3h synchronously with figure 1B–D. The amount of *fepA*, *fecB* and *fhuF* mRNA was detected by qRT-PCR and compared with 0h. All the values shown represent mean ± standard deviation from three independent experiments.

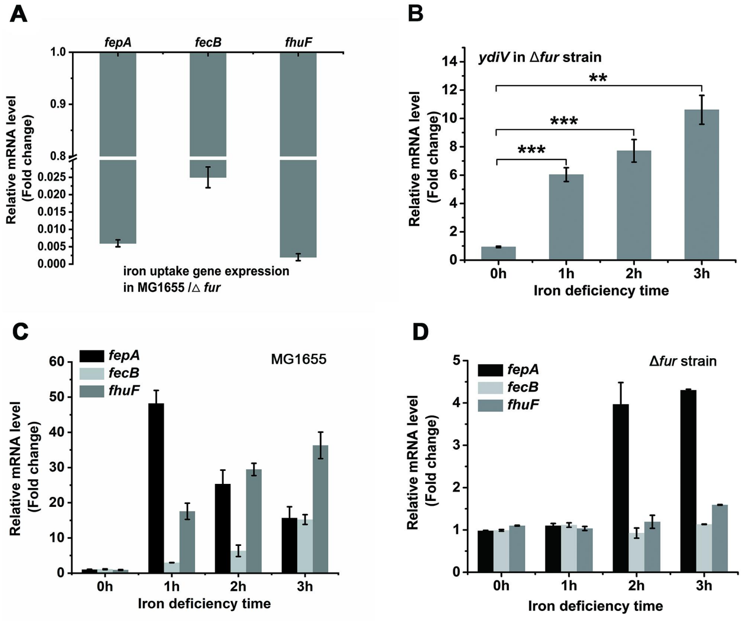

Supplementary Figure S3. The mRNA level of *ydiV* and iron uptake genes in MG1655 or Δ*fur* strains**.** (**A**) In LB medium, the mRNA level of iron uptake system genes in MG1655 in comparison with that in Δ*fur* strain were detected by qRT-PCR. (**B**) The mRNA level of *ydiV* in Δ*fur* strain before (0h) and after (1-3h) facing iron-limited environment was detected by qRT-PCR. The expression of *ydiV* under iron deficiency in comparison with that before iron deficiency (0h) using the *t*-test. **, $P < 0.01$; ***, $P < 0.001$. (**C–D**) The iron uptake genes transcription in MG1655 and Δ*fur* strain during iron deficiency induction is monitored by qRT-PCR. 200 μM 2,2'-dipyridyl was added in LB medium for generating iron-limited condition. All the values shown represent mean ± standard deviation from three independent experiments.

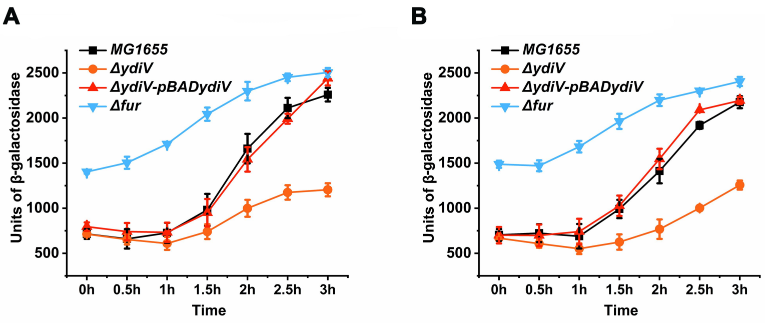

Supplementary Figure S4. The *fepA* promoter (**A**) and *fhuF* promoter (**B**) activity in iron sufficient condition was tested by β-galactosidase assays. The target strain was inoculated into LB medium and cultured until OD_{600}=0.2. Then 0.8 mg/ml arabinose was added to activate *ydiV* gene expression. Cultures at different induction times were reserved to detect

β-galactosidase activity. The values shown as represent mean ± standard deviation from three independent experiments.

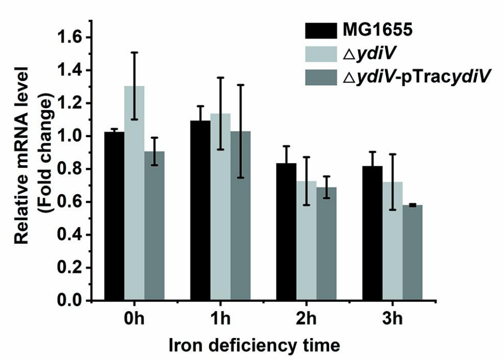

Supplementary Figure S5. The expression of *fur* in strains with different levels of YdiV. The mRNA level of *fur* gene in MG1655, Δ*ydiV* and Δ*ydiV*-pTracy*diV* strains before (0h) and after (1–3h) facing iron-limited environment was detected by qRT-PCR. Three biological replicates were performed for each sample.

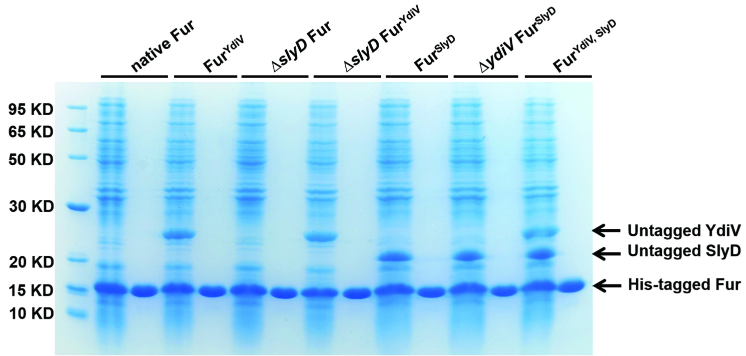

Supplementary Figure S6. The SDS-PAGE for native Fur and Fur co-expressed with YdiV or SlyD. Protein names are marked on the top of the picture. For each set of samples, the first lane represents the whole cell lysate of the protein-expressing strain, the second lane represents the protein purified after Ni-NTA affinity column and Size-Exclusion chromatography.

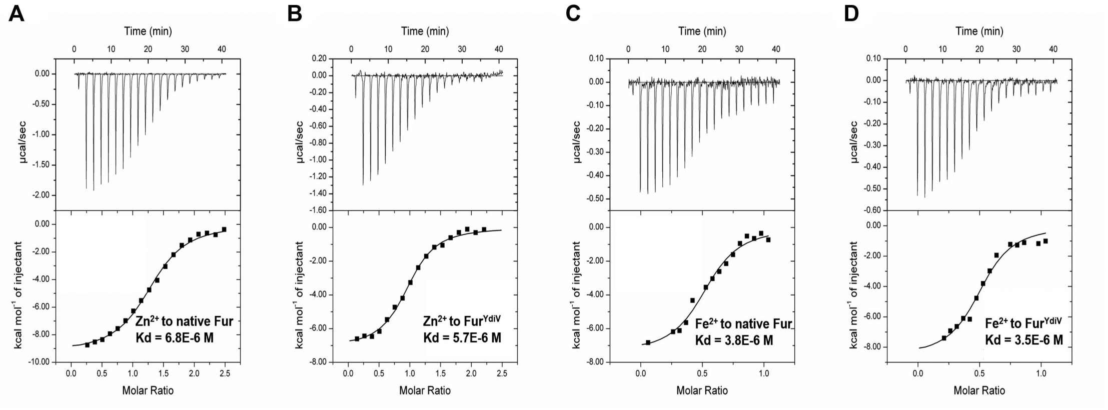

Supplementary Figure S7. Characterization of binding affinities of native Fur and Fur^{YdiV} for metal cofactors. The dissociation constant of Zn^{2+} with native Fur (**A**), Zn^{2+} with Fur^{YdiV} (**B**),

Fe^{2+} with native Fur (**C**) and Fe^{2+} with Fur^{YdiV} (**D**) was detected by ITC and marked in the corresponding curve.

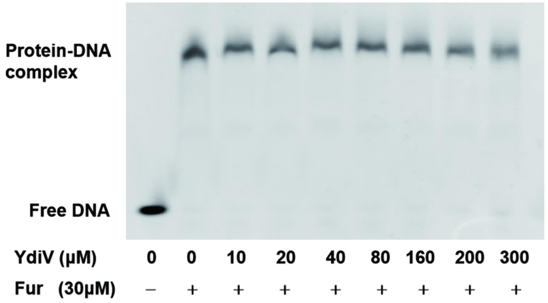

Supplementary Figure S8. EMSA for native Fur and Fur box DNA with different concentrations of YdiV. 30 μM of Fur was mixed with different concentrations of YdiV for 10 min and then the DNA-binding ability was examined by EMSA with 25 nM FAM-labeled Fur box DNA. The amount of YdiV added in this experiment was annotated in the figure. The experiment was repeated three times with similar results.

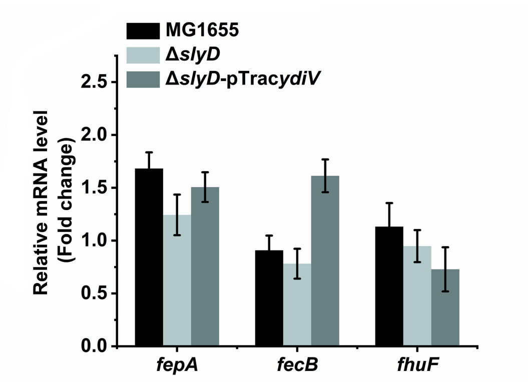

Supplementary Figure S9. The expression of iron uptake genes in MG1655, Δ*slyD* and Δ*slyD*-pTrac*ydiV* strains during iron sufficiency. The mRNA level of *fepA*, *fecB* and *fhuF* was monitored by qRT-PCR. Strains were cultured in LB medium to OD_{600}=0.6, then IPTG was added for *ydiV* induction. Samples were collected at 3h after induction. Three biological replicates were performed.

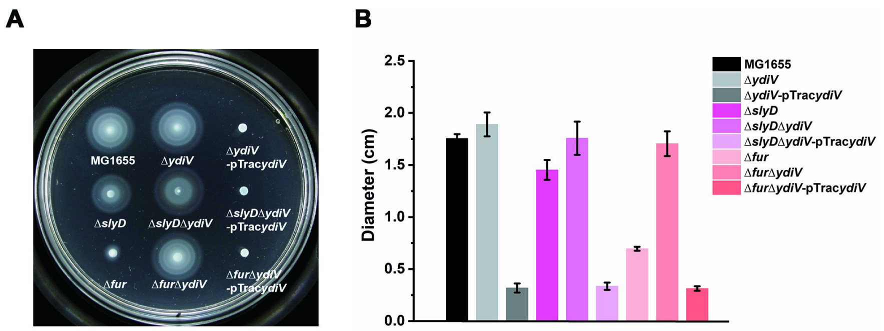

Supplementary Figure S10. The inhibition of flagella synthesis by YdiV in WT, Δ*fur* and Δ*slyD* strains. (**A**) 0.5 ul overnight bacterial culture was inoculated on the LB plate containing 0.2%

agar. (**B**) The diameters of bacterium colonies. Four biological replicates were performed for each sample.

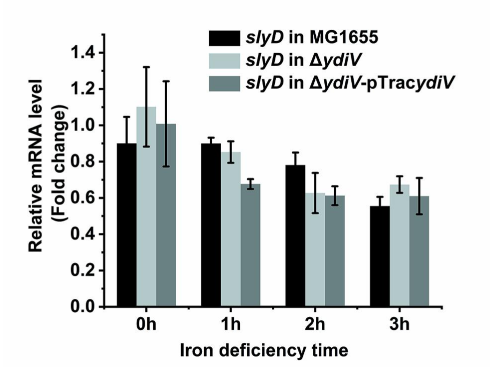

Supplementary Figure S11. The expression of *slyD* in strains with different levels of YdiV. The mRNA level of *slyD* gene in MG1655, Δ*ydiV* and Δ*ydiV*-pTrac*ydiV* strains before (0h) and after (1–3h) facing iron-limited environment were detected by qRT-PCR. Three biological replicates were performed for each sample.

Supplementary Table S1. Bacterial strains used in this study.

E.coli strain	Relevant characteristics	Source
DH5α	A host for plasmid cloning.	Our lab
MG1655	Strain K-12, F^{-} λ^{-} *rph-1.*	Our lab
MG1655 Δ*ydiV*	*ydiV* deletion mutant of MG1655.	This study
MG1655 Δ*slyD*	*slyD* deletion mutant of MG1655.	This study
MG1655 Δ*fur*	*fur* deletion mutant of MG1655.	This study
BL21(DE3)	F′ *ompT* $hsdS_B$ (rB^{-} m_B^{-}) *gal dcm met* (DE3).	Our lab
BL21 Δ*ydiV*	*ydiV* deletion mutant of BL21(DE3).	This study
BL21 Δ*slyD*	*slyD* deletion mutant of BL21(DE3).	This study
BL21 Δ*fur*	*fur* deletion mutant of BL21(DE3).	This study
UPEC CFT073	Wild type.	Shigan Yan[a]
UPEC Δ*ydiV*	*ydiV* deletion mutant of UPEC CFT073.	This study
UPEC Δ*slyD*	*slyD* deletion mutant of UPEC CFT073.	This study
BTH101	F′, *cya*-99, *araD139*, *galE15, galK16, rpsL1*(Str^R), *hsdR2, mcrA1, mcrB1, relA1.*	Beile Gao[b]

[a]Qilu University of Technology. [b]Chinese Academy of Sciences, South China Sea Institute of Oceanology.

Supplementary Table S2. Oligonucleotides used in this study for gene knockout. The letters with underline represented homologous sequences for recombination.

Oligonucleotides	Sequence
Gene knockout	
ydiV-Kan-F	ATCCCTTTTAGCCGGATACTGAAAAACATCCTTCGAGAGGGACGGTTACCGTGTAGGCTGGAGCTGCTTC
ydiV-Kan-R	TTGATAGTGTAGACGGTTACTCTCGTCTTAAACACCAGCAAACAGAAGGGATGGGAATTAGCCATGGTCC
slyD-Kan-F	TTAAGCTAGTGAGTACACGGCTGCAGAATTCCGCTACAATCTGCGCCACTATTCTTCCCATGCTCAGGAGATATCGTGTAGGCTGGAGCTGCTTC
slyD-Kan-R	CGGTATTAGTGGCAACCGCAACCGCCGTTGCCTTTACCGCCACAGCAGCCTTCGCCACCGTGTTCATGACCGTGAATGGGAATTAGCCATGGTCC
fur-Kan-F	TGTCACTTCTTCTAATGAAGTGAACCGCTTAGTAACAGGACAGATTCCGCGTGTAGGCTGGAGCTGCTTC
fur-Kan-R	CTTGCATAAAAAAGCCAACCCGCAGGTTGGCTTTTCTCGTTCAGGCTGGCATGGGAATTAGCCATGGTCC
Gene test	
ydiV-test-F	GCCCCGTGAAACCAAACAAC
ydiV-test-R	GCCTCACTGGCGAGACTTT
slyD-test-F	ATATGCGGATGCTGCGAGAG
slyD-test-R	CATATCTCCAGGATCGGGGC
fur-test-F	GCGGGCTATCATTTCGAAGC
fur-test-R	ACCATCTTCTGATGCCCGAC

Supplementary Table S3. Plasmids used in this study.

Plasmids	Relevant characteristics	Source
pTKRED	Gene knockout plasmid, expression of three λ-Red recombination enzyme.	Qingsheng Qi[a]
pCP20	Resistance gene remove plasmid, expression of flippase recombination enzyme.	Qingsheng Qi[a]
pKD4	Amplification the kanamycin gene cassettes flanked by FRT recognition target sites.	Qingsheng Qi[a]
pTrac99a	Cloning vector, *trc* promoter, *ColE1* ori, *Amp.*	Qingsheng Qi[a]
pTrac*ydiV*	*ydiV* from MG1655 cloned into pTrac99a vector.	This study
pBAD24	Cloning vector, *araBAD* promoter, *f1* ori, *Amp.*	Our lab
pBAD*ydiV*	*ydiV* from MG1655 cloned into pBAD24 vector.	This study
pCL1920	Cloning vector, *lac* promoter, *pSC101* ori, *spc.*	Qingsheng Qi[a]
pCL *fepAp-lacZ*	*fepA* promoter from MG1655 and *lacZ* cloned into pCL1920 vector instead of *lac* promoter.	This study
pCL *fhuFp-lacZ*	*fhuF* promoter from MG1655 and *lacZ* cloned into pCL1920 vector instead of *lac* promoter.	This study
pGL01	Expression vector, *T7 lac* promoter, N-terminal His tag, PPase cleavage site between the His-tag and the expressed protein, Amp.	Our lab
pGL01*ydiV*	YdiV protein expression construct into pGL01 vector, N-terminal His tag.	This study
pGL01*slyD*	SlyD protein expression construct into pGL01 vector, N-terminal His tag.	This study
pGL01*fur*	Fur protein expression construct into pGL01 vector, N-terminal His tag.	This study
pGL01*fur* P18A	Fur P18A mutant protein expression construct into pGL01 vector, N-terminal His tag.	This study
pGL01*fur* P29A	Fur P29A mutant protein expression construct into pGL01 vector, N-terminal His tag.	This study
pACYCDuet-1	Expression vector, *T7 lac* promoter, N-terminal His tag, *cmr.*	Our lab
pACYC*ydiV*	YdiV protein expression construct into pACYCDuet-1 vector, no tag.	This study
pET29b	Expression vector, *T7 lac* promoter, c-terminal His tag, *Kan.*	Our lab
pET29b*slyD*	SlyD protein expression construct into pET29b vector, no tag.	This study
p*ydiV*	*ydiV* from UPEC cloned into pCL1920 vector.	This study
p*slyD*	*slyD* from UPEC cloned into pCL1920 vector.	This study
pKT25	*lac* promoter, *p15A* ori, N-terminal T25, *Kan.*	Beile Gao[b]
pUT18C	*lac* promoter, *ColE1* ori, N-terminal T18, *Amp.*	Beile Gao[b]
pKNT25	*lac* promoter, *p15A* ori, C-terminal T25, *Kan.*	Beile Gao[b]
pCH363	*lac* promoter, *pBR322* ori, C-terminal T18, *Amp.*	Beile Gao[b]

pKT25-zip	The leucine zipper region of the GCN4 from yeast cloned into pKT25 vector. Positive control.	Beile Gao[b]
pUT18C-zip	The leucine zipper region of the GCN4 from yeast cloned into pUT18C vector. Positive control.	Beile Gao[b]
pKNT25-*slyD*	*slyD* from *MG1655* cloned into pKNT25 vector.	This study
pKNT25-*ydiV*	*ydiV* from *MG1655* cloned into pKNT25 vector.	This study
pCH363-*ydiV*	*ydiV* from *MG1655* cloned into pCH363 vector.	This study
pUT18C-*fur*	*fur* from *MG1655* cloned into pUT18C vector.	This study

[a]Shandong University. [b]Chinese Academy of Sciences, South China Sea Institute of Oceanology.

Supplementary Table S4. Oligonucleotides used in this study for plasmid construction. The letters with underline represented homologous sequences for recombination.

Oligonucleotides	Sequence
pTrac99a-F	CGAGAGTAGGGAACTGCCAG
pTrac99a-R	GGTCTGTTTCCTGTGTGAAATTG
pTrac*ydiV*-F	AACAATTTCACACAGGAAACAGACCATGAAGATTTTTTTGGAGAATCTTTATC
pTrac*ydiV*-R	ATGCCTGGCAGTTCCCTACTCTCGTTATCGCTGAACCAACGTCGTTATCTG
pBAD24-F	AAGCTTGGCTGTTTTGGCGGATGAG
pBAD24-R	GAATTCCTCCTGCTAGCCCAAAAAAACG
pBAD*ydiV*-F	TTTTTTGGGCTAGCAGGAGGAATTCATGAAGATTTTTTTGGAGAATCTTTATC
pBAD*ydiV*-R	CTCATCCGCCAAAACAGCCAAGCTTTTATCGCTGAACCAACGTCGTTATCTG
pCL1920-Np-F	TAAGCCAGCCCCGACACC
pCL1920-Np-R	ACGCGAATTCCCGACAGTAAGA
pCL *fepAp*-F	TTACTGTCGGGAATTCGCGTACCATAACCCCATGTTTACTGTG
pCL *fepAp*-R	GAATCCGTAATCATGGTCATTGTTTTATTCCTGCATTTTTGCCAC
pCL *fhuFp*-F	TTACTGTCGGGAATTCGCGTAATCCGCCACTGATCTGACG
pCL *fhuFp*-R	GAATCCGTAATCATGGTCATAATCGGGATAGTAATCTAAATGATAATG
pCL *lacZ*-F	GGCAAAAATGCAGGAATAAAACAATGACCATGATTACGGATTCACTGG
pCL *lacZ*-R	CGGGTGTCGGGGCTGGCTTATTATTTTTGACACCAGACCAACTGG
pGL01-F	CTCGAGTCTGGTAAAGAAAC
pGL01-R	GGATCCGGGCCCCTGGAA
pGL01*ydiV*-F	TTCCAGGGGCCCGGATCCATGAAGATTTTTTTGGAGAATCTTTATC
pGL01*ydiV*-R	TTCTTTACCAGACTCGAGTTATCGCTGAACCAACGTCGTTATCTG
pGL01*slyD*-F	TTCCAGGGGCCCGGATCCATGAAAGTAGCAAAAGACCTGGTG
pGL01*slyD*-R	TTCTTTACCAGACTCGAGTTAGTGGCAACCGCAACCG
pGL01*fur*-F	TTCCAGGGGCCCGGATCCATGACTGATAACAATACCGCCCT
pGL01*fur*-R	TTCTTTACCAGACTCGAGTTATTTGCCTTCGTGCGCATGTT
pGL01*fur*-P18A-F	CCTGAAAGTAACGCTTGCCCGTTTAAAAATCCTGGAAGTTCTTCAGG

pGL01*fur*-P18A-R	GGCAAGCGTTACTTTCAGGCCAGCTTTCTTTAGGGCG
pGL01*fur*-P29A-F	GGCCGACAACCATCACGTCAGTGCGGAAGATTTATACAAACG
pGL01*fur*-P29A-R	ACGTGATGGTTGTCGGCCTCCTGAAGAACTTCCAGGATTTTTAAAC
pACYCDuet-1-F	TAATTAACCTAGGCTGCTGCCAC
pACYCDuet-1-R	GGTATATCTCCTTATTAAAGTTAAACAAAATTATTTCTAC
pACYC*ydiV*-F	TTTAACTTTAATAAGGAGATATACCATGAAGATTTTTTTGGAGAATCTTTATC
pACYC*ydiV*-R	GTGGCAGCAGCCTAGGTTAATTATTATCGCTGAACCAACGTCGTTATCTG
pET29b-F	CTCGAGCACCACCACCACCACCACTGAGATCCGGCTGCTAACAAAGC
pET29b-R	CATATGTATATCTCCTTCTTAAAGTTAAACAAAATTATTTCTAGAGGGGAATT
pET29b*slyD*-F	GAAGGAGATATACATATGAAAGTAGCAAAAGACCTGGTG
pET29b*slyD*-R	GTGGTGGTGGTGCTCGAGTTAGTGGCAACCGCAACCG
pCL1920-F	TAAGCCAGCCCCGACACC
pCL1920-R	AGCTGTTTCCTGTGTGAAATTGTTA
pCL1920*ydiV*-F	ATTTCACACAGGAAACAGCTATGAAGATTTTTTTGGAGAATCTTTATC
pCL1920*ydiV*-R	CGGGTGTCGGGGCTGGCTTATTATCGCTGAACCAACGTCGTTATCTG
pCL1920*slyD*-F	ATTTCACACAGGAAACAGCTATGAAAGTAGCAAAAGACCTGGTG
pCL1920*slyD*-R	CGGGTGTCGGGGCTGGCTTATTAGTGGCAACCGCAACCG
pUT18C-F	TAATATGGTGCACTCTCAGTACAATCTGCTC
pUT18C-R	CTCGAGATCTAGAGTCGACCTGCA
pUT18C-*fur*-F	GGTCGACTCTAGATCTCGAGATGACTGATAACAATACCGCCCT
pUT18C-*fur*-R	ACTGAGAGTGCACCATATTA TTATTTGCCTTCGTGCGCATGTT
pKNT25-F	TGCAGGTCGACTCTAGAGGATCCC
pKNT25-R	GGCATGCAAGCTTGGCGTAAT
pKNT25-*slyD*-F	ACGCCAAGCTTGCATGCCATGAAAGTAGCAAAAGACCTGGTG
pKNT25-*slyD*-R	TCCTCTAGAGTCGACCTGCA GTGGCAACCGCAACCG
pKNT25-*ydiV*-F	ACGCCAAGCTTGCATGCCATGAAGATTTTTTTGGAGAATCTTTATC
pKNT25-*ydiV*-R	TCCTCTAGAGTCGACCTGCA TCGCTGAACCAACGTCGTTATCTG
pCH363-F	ATTACGCCAAGCTTGCATGCC
pCH363-R	CATGGTCATAGCTGTTTCCTGTGTG
pCH363-*ydiV*-F	AGGAAACAGCTATGACCATGATGAAGATTTTTTTGGAGAATCTTTATC
pCH363-*ydiV*-R	GCATGCAAGCTTGGCGTAATTCGCTGAACCAACGTCGTTATCTG

Supplementary Table S5. Primers used in real-time quantitative PCR.

Gene	Forward sequence (5'→3')	Reverse sequence (5'→3')
gapA	AACTGAATGGCAAACTGACTGGTA	TTTCATTTCGCCTTCAGCAGC
fur	ACTGACACAGCAACATCACCACG	ACTGTGGTTAGTCAGGCGAATGC
ydiV	GCAACTTACCGAGGAGCAACAT	CCTTCATTGAGATGCGGGTAA
slyD	TCACTGCGGTTGAAGACGAT	TGAACGTGACCATGAGCCAG
fepA	AACCTGACCGGTAACTCCACCA	GGCACCCAGGAAGTATCACCAC
fhuF	TGCTGGAGTTTATCCGCCTG	TCATCATCGGTTGGTTGCGA
fecB	CCCACTATCGCGAAGAGAGCAT	TAAGCGGCTGATGGTGGAAGAT

Supplementary Table S6. Representative MS analysis of His-Fur pull-down.

Accession	Gene	Coverage	Peptides	Gene product or Function
A0A0G3HUH8	*fur*	93.91	20	Ferric iron uptake regulon transcriptional repressor.
A0A0G3HNG0	*ydiv*	72.99	12	Anti-$FlhD_4C_2$ factor, inactive EAL family protein.
A0A0G3HX96	*flhd*	97.41	10	Flagellar transcriptional regulator.
A0A0S1EZY8	*slyD*	35.71	4	Peptidyl-prolyl cis-trans isomerase.
P0ACJ8	*crp*	41.42	8	cAMP-activated global transcriptional regulator.
A0A0G3I191	*fbaA*	18.66	4	Class II fructose-bisphosphate aldolase.
P27250	*ahr*	8.55	2	Aldehyde reductase Ahr.
A0A0G3I0D1	*rplN*	26.82	3	50S ribosomal protein L14.
P0A7L8	*rpmA*	29.41	2	50S ribosomal protein L27.
P64588	*yqjI*	18.35	4	Transcriptional regulator YqjI.
P0A991	*fbaB*	18.57	5	Fructose-bisphosphate aldolase class 1.
P0A836	*sucC*	7.21	3	Succinyl-CoA ligase [ADP-forming] subunit beta.
A0A0G3HML4	*rpmB*	24.35	2	50S ribosomal protein L28.
A0A0G3HMH5	*gatD*	9.53	3	Galactitol-1-phosphate 5-dehydrogenase.
P06610	*btuE*	18.03	2	Vitamin B12 transport periplasmic protein BtuE.
P62399	*rplE*	22.34	3	50S ribosomal protein L5.
